# Walknet, a bio-inspired controller for hexapod walking

**DOI:** 10.1007/s00422-013-0563-5

**Published:** 2013-07-04

**Authors:** Malte Schilling, Thierry Hoinville, Josef Schmitz, Holk Cruse

**Affiliations:** Department of Biological Cybernetics and Theoretical Biology, Bielefeld University, P.O. Box 100131, 33501 Bielefeld, Germany

**Keywords:** Insect locomotion, Motor control, Decentralized architecture

## Abstract

Walknet comprises an artificial neural network that allows for the simulation of a considerable amount of behavioral data obtained from walking and standing stick insects. It has been tested by kinematic and dynamic simulations as well as on a number of six-legged robots. Over the years, various different expansions of this network have been provided leading to different versions of Walknet. This review summarizes the most important biological findings described by Walknet and how they can be simulated. Walknet shows how a number of properties observed in insects may emerge from a decentralized architecture. Examples are the continuum of so-called “gaits,” coordination of up to 18 leg joints during stance when walking forward or backward over uneven surfaces and negotiation of curves, dealing with leg loss, as well as being able following motion trajectories without explicit precalculation. The different Walknet versions are compared to other approaches describing insect-inspired hexapod walking. Finally, we briefly address the ability of this decentralized reactive controller to form the basis for the simulation of higher-level cognitive faculties exceeding the capabilities of insects.

## Control of walking

The fundamental task of a brain is to allow an organism for controlling active locomotion (e.g., Wolpert et al. 2001). Comparing the three basic types of active locomotion, swimming, flying, and walking, the latter is presumably the most complex one with respect to controllability, making the investigation of the control structure of such a system a challenging task. Due to its complexity, a system that is able to control multi-legged walking does not suit well the approach applied in traditional physics or in physiology, for example. The latter systems are characterized by a clearly definable input and a measurable output which together can be used for system identification. In contrast, a walking system is characterized by a high number of degrees of freedom. Here we focus on a six-legged insect (or robot) with three active joints per leg that is characterized by at least 18 degrees of freedom^1^ and a large number of sensory input channels. Two properties make such a system differing from those studied traditionally. First, due to the redundancy of both the effectors and the information given by sensory input, there is no unique solution of how to respond to a given physical situation. The controller has to select one out of numerous possible solutions and therefore forms an underdetermined system. This means that the controller has to make autonomous decisions when adapting to the current context. The issue of how to deal with redundancy concerns not only decisions with respect to the motor output, but also with respect to the interpretation of redundant sensory inputs. Second, the system is even less determined as its behavior strongly depends on the feedback from the environment to which the body is mechanically coupled. As this coupling—the “loop through the world,” i.e., body including muscles plus environment—may be exploited by the system to simplify the necessary neuronal computation (for a striking example see Schmitz et al. 2008), properties of the unpredictable environment have to be considered as part of the properties of the complete system making the study of such systems even more challenging.

Therefore, an adequate research strategy to study such a system is to follow a holistic approach. This means that the complete biological system, i.e., the intact animal, has to be studied as it behaves in various situations as freely as possible. Such studies may lead to quantitative hypotheses in the form of software simulations as well as hardware simulations (i.e., robots). Of course, this holistic approach has to be paralleled by traditional physiological studies investigating subsections of the complete system, because such studies allow for decreasing the number of possible hypotheses. Following the latter, traditional, approach alone may, however, not easily lead to an understanding of the whole system. Together, the two approaches can complement each other. Physiological studies provide insights into concrete structural questions, whereas a holistic approach allows for the understanding of emergent properties as well as for posing new questions to be studied on the physiological level.

In this article, we deal with an artificial neural network, Walknet, that has been developed to describe the principles underlying hexapod walking as it can be observed in insects, in particular in stick insects. Although using artificial neurons as structural elements, Walknet, being a phenomenological model, should not be understood as a model describing the neuronal architecture itself, but as a quantitative, consistent hypothesis summarizing behavioral findings. Nonetheless, Walknet might later be detailed by replacing specific sections with biologically more realistic neuronal structures, in this way approaching another level of description that is accompanied by a considerable increase of possible degrees of freedom. As it stands, Walknet, although it represents a simple reactive system, is able to describe quite complex behavioral sequences as are for example required to climb over a very large gap (Bläsing 2006). This is possible because Walknet is constructed of a (large) number of simple procedural modules that may act in parallel or may compete for access of the motor output. Autonomy of the system, in the sense of being able to select between different behaviors, is reached by the introduction of an overarching network consisting of so-called motivation units.

**Fig. 1:**
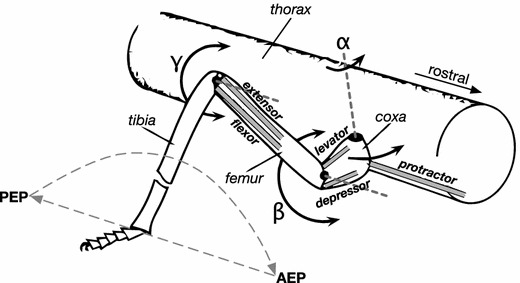
Schematic diagram showing the morphology of a stick insect leg. Angle $\upalpha$ describes the position of the Thorax-Coxa joint (muscles Protractor-Retractor), angle $\upbeta$ stands for the position of the Coxa-Trochanterofemur joint (muscles Levator-Depressor), and angle $\upgamma$ describes the position of the Femur-Tibia joint (muscles Flexor-Extensor). The axis of rotation of the Thorax-Coxa joint is defined by angles $\upphi$ and $\uppsi$ relative to the body-fixed coordinate system (only the vertical axis *z* is marked). Swing movement and stance movement are sketched by dashed lines. *AEP* anterior extreme position, *PEP* posterior extreme position

**Fig. 2:**
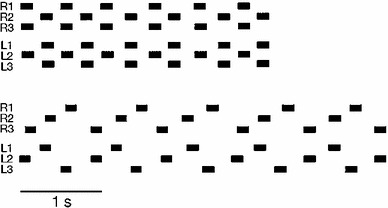
Typical examples of tripod (*above*) and tetrapod (*below*) gait (redrawn after Graham 1972). Abscizza is time, *black bars* indicate swing movement. R1, R2, R3 right front, middle, and hind legs, respectively. L1, L2, L3: corresponding left legs

Before explaining details, some basic terminology should be introduced. On the phenomenological level, a walking leg can be characterized to be in one of two states, “*swing*” or “*stance*” (sometimes called return stroke and power stroke, respectively). During the swing movement, the leg is lifted off the ground and moved to a position where the next stance movement can be started. During a stance movement, the body is supported and moved in the desired direction. Swing and stance movements are usually characterized by two positions, defined in a body-fixed coordinate system (Bässler 1972). The *posterior extreme position* (PEP) is defined as the position at which the leg is lifted off the ground to start a swing movement. The *anterior extreme position* (AEP) defines the position where the leg switches from swing to stance by touching the ground (see Fig. 1). The cooperation of the legs results in spatio-temporal walking patterns which were, by earlier authors (e.g., Graham 1972, 1985; Hughes 1952; Wilson 1966), defined by the terms *tripod* gait, *tetrapod* gait, and *wave* gait (further names have been used, too). Figure 2 depicts a typical tripod pattern and a typical tetrapod pattern. Loosely defined, in tripod at least three legs, in tetrapod at least four legs, and in wave gait at least five legs are on the ground at any time (for attempts of more quantitative definitions see Wosnitza et al. 2013; Grabowska et al. 2012). Although generally used, the term “gait” may be, however, misleading as, in insects, there are no fixed patterns with instable transitions as found in walk, trot, and gallop of horses, for example (see Graham 1972, Fig. 7). Instead, there exists a continuum of phase relations between the legs.

To avoid a possible confusion always possible when simulating animal behavior by artificial neural networks, we will talk of the neuronal system/unit when addressing the biological neurons underlying the behavior and of the neural system/unit when addressing how the simulation is implemented.

In Sect. 2 of this article, we briefly report on earlier models that can be considered as precursors to Walknet. In Sect. 3, we describe the different versions of Walknet. Over the years, Walknet was subject to changes in detail to cover specific aspects. As will be reported, some of these aspects have been simulated separately but not (yet) implemented in any complete version of Walknet. In Sect. 4, we will briefly review the most important results that have been obtained studying the walking behavior of insects, in particular stick insects, which together with cockroaches (Beer et al. 1993, 1997; Ritzmann and Büschges 2007), are the most intensively studied insects in this area. In this section, we will show to what extent Walknet can simulate these data gained from a huge number of biological experiments. Section 5 deals with open questions and with related work. Further assumptions will be introduced in Sect. 6 explaining to what extent this simple reactive system can form the basis for higher cognitive function. While these assumptions are not supported by results found on the insect level, they are taken from studies made with other, “higher” animals including humans. Along these lines, Walknet, as such forming a reactive and embodied system, can serve as a starting point for further expansions, following an evolutionary path to higher-level function, being able to simulate cognitive abilities.

## Development of Walknet, precursor models

Walknet has, of course, precursors. All precursor models of Walknet describing the gait pattern of walking insects and published between 1960 and 1980 consider simplified legs showing only one joint which allows to represent protraction and retraction.

Inspired by ideas of von Holst to understand gliding coordination, Wendler (1964) and Graham (1977) developed models in which each leg is characterized by a relaxation oscillator. The biological interpretations of these oscillators are not further specified and could be interpreted as to represent a reflex chain or a central oscillator. Coupling of the oscillators in the model of Wendler is done by continuous analog coupling signals, i.e., signals that are not restricted to a temporal window during the step cycle. Wendler’s model assumes a mutual coupling between all neighboring legs of the same body segment, i.e., between contralateral legs, as well as coupling from rear to front between all ipsilateral neighbors, but also from hind leg to front leg. In Graham’s model, two relaxation oscillators describing two neighboring legs are coupled via a delay oscillator acting in both directions between contralateral neighboring legs and from rear to front between ipsilateral legs. The delay oscillator is inspired by Wilson (1966) who postulated a coordination influence very similar to the one which was later called rule 1 (e.g., Dürr et al. 2004, and below, Sect. 3). In Graham’s model, the delay is controlled by a central command that also influences the leg oscillators and represents walking velocity. Both models are able to simulate walking with different velocities showing smooth, stable transitions between slow (“tetrapod”) and fast (“tripod”) gaits. Wendler’s model can show a changed phase shift between hind legs and front legs as observed by Wendler (1964) when middle legs of the insect are amputated.

Although being of simple structure, the model of Pearson and Iles (1973) makes more detailed assumptions concerning the architecture of the leg controller. In this model, each leg controller is described by two neuron-like units, an autonomously oscillating Levator unit (for swing) which when active inhibits activation of the other, the Depressor unit (representing stance movement). Levator units of neighboring legs inhibit each other, the connectivity being restricted to simulate tripod gait patterns.

In contrast to Graham’s (1977) model, the model of Cruse (1979a, b) used relaxation oscillators with different speed for swing and stance movements. A coupling influence triggered the stance-swing transition depending on the position of the anterior leg. When the anterior leg is in stance, the PEP of the influenced leg is changed. Qualitatively this influence corresponds to what later has been termed rule 3 (e.g., Dürr et al. 2004, and below, Sect. 3). In addition, there are two coupling influences connecting diagonally neighboring legs, for which no experimental evidence has been found up to now. In a later model of Cruse (1980a, b), the leg controller is principally the same, but is more detailed concerning the sensory feedback (position and load). The model, although basically forming a sensory feedback system, allows for motor output being driven by a central oscillator when sensory feedback is decreased [an approach further studied by Beer and Gallagher (1992), Gallagher and Beer (1993) and recently by Daun-Gruhn (2011), see also Ijspeert (2008) for references]. Four local coordinating influences are hypothesized. Three of them qualitatively correspond to later defined coordination rules 1, 3, and 5 (see Sect. 3), the fourth is the diagonal connection mentioned above for which no experimental support has been found. The properties of the model have been tested on a large number of experimental findings available in the late 1970s.

## Walknet

**Fig. 3:**
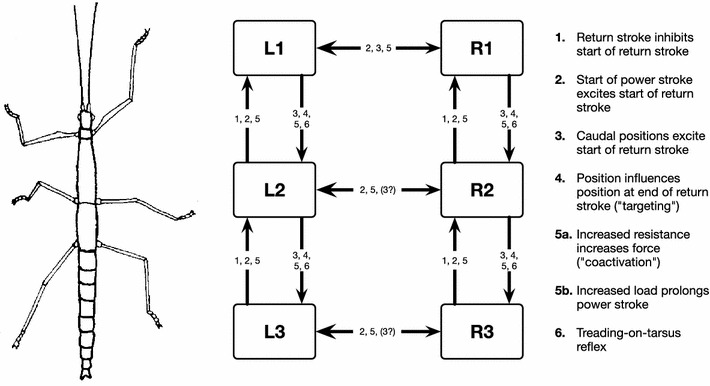
Leg modules and their connection via coordination rules (from Dürr et al. 2004). L1, L2, L3 left front, middle, and hind leg, respectively. R1, R2, and R3 stand for the corresponding right legs. The question mark indicates that there are ambiguous data concerning this influence

As a successor of the Cruse (1980a, b) model, Walknet has originally been developed as a network that is able to control the movement of a 18 degrees of freedom (DoF) system, which consists of a rigid body with six legs, each showing three active joints ($\upalpha$-, $\upbeta$-, and $\upgamma$-joint, Fig. 1). The architecture of Walknet basically contains six in principle independent controllers, one for each leg (Fig. 3). These controllers are connected in three ways.

(i) Leg controllers are connected “horizontally” via the coordination rules (influencing PEPs and AEPs), as well as (ii) “vertically” coupled via a heterarchical network, the so-called motivation unit net. Furthermore, (iii) legs are mechanically coupled via the substrate and the body which, via sensory feedback, influences the controller of the other legs. As each of these three connection layers—the mechanical, the motivational and the coordination rule layer—forms a recurrent network by itself, we deal with a highly complex dynamical system.

**Fig. 4:**
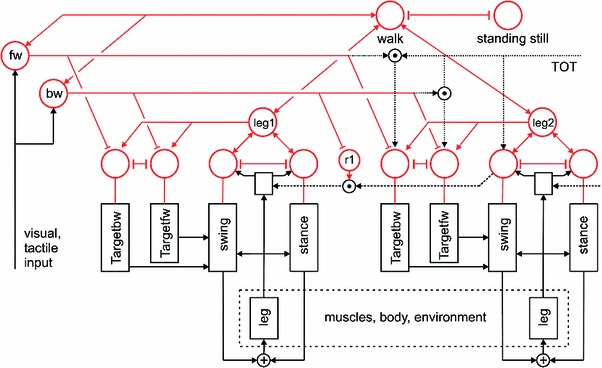
The general architecture of Walknet. Only two leg controllers are shown (for details see Fig. 5). The upper part contains the motivation units (all marked in *red*) forming a heterarchical network influencing the procedures (*black boxes*, e.g., Swing-net, Stance-net, Target-net_fw, and Target-net_bw, representing the end point of the swing movement for forward walking and backward walking, respectively). Furthermore, there are “higher-level” motivation units as leg1, walk, as well as forward (fw) and backward (bw). A motivation unit able to control a coordination influence between leg1 and leg 2 is marked by r1. Motivation units form a recurrent neural network coupled uni- or bidirectionally by positive (*arrowheads*) and negative (T-shaped connections) influences. The lower part of the figure (*dashed box* muscles/body/environment) schematically depicts the “loop through the world”

Each of the controllers consists of several procedural elements, essentially a Swing-net, a Stance-net, and Target-nets as depicted in Fig. 4, where only two of the six controllers are depicted. Stance-net controls the stance movement, Swing-net controls the trajectory of the swing movement, the Target-nets contain information concerning the end position of a swing movement used by the corresponding Swing-net. In general, parameters in the Walknet versions, i.e., the weights of the neural networks are either optimized by hand tuning or learned off-line to match the observed behavioral data.

Over the years, Walknet was subject to changes in detail to cover specific aspects. Some procedures have up to now only been tested in isolated software simulations but not (yet) implemented in any complete version of Walknet, to keep complexity in a manageable range. In the actual implementation of Walknet residing on the robot Hector (Paskarbeit et al. 2010; Schneider et al. 2011), several of these versions are implemented in parallel. In this way, they can be tested separately.

The general architecture of Walknet is illustrated in Fig. 4. The upper part (in red) shows the motivation units, below them the procedures (black boxes). The body (plus environment) is depicted by a dashed box. We will first describe the leg controller, which will be followed by an explanation of the coordination rules. We will then focus on the motivation unit network and finally briefly characterize the simulation of the body in the different versions of Walknet.

### Leg controller

**Fig. 5:**
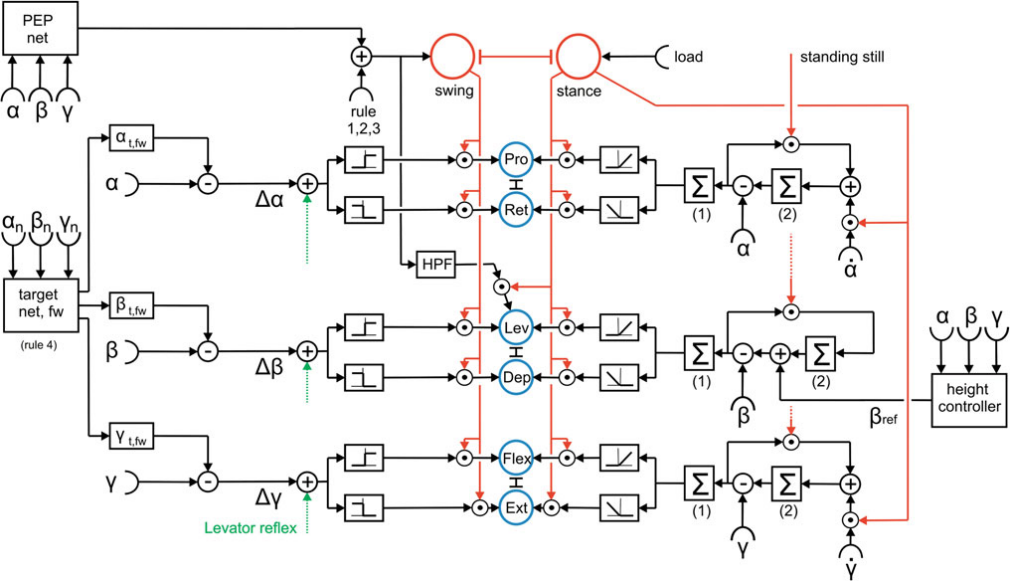
A network diagram describing a leg controller that summarizes a number of behavioral observations as detailed in the text. The right hand side depicts the sensory input relevant for stance control, the left hand side correspondingly the sensory input required for the control of the swing movement. Pairs of units depicted in *blue* at the center (Protractor-Retractor, Levator-Depressor, Flexor-Extensor) which are coupled via mutual inhibition (T-shaped connections), represent an abstract version of the network shown by Schumm and Cruse (2006, their Fig. 7). For definition of joint angles $\upalpha , \upbeta$, and $\upgamma$ see Fig. 1. Two motivation units (swing, stance, marked in *red*) control the sensory input to the control network via inhibitory connections

As sketched in Fig. 4, the leg controller consists of several functional elements, the most important procedures being Stance-net, Swing-net, and Target-net. As these procedures are functionally defined, not morphologically, in Fig. 4, they are plotted as separate boxes. In contrast to earlier statements (Daun-Gruhn and Büschges 2011), this functional separation does not mean that the Walknet architecture requires a neuronal separation of units belonging to a network exclusively obeying to a Swing controller or a Stance controller. Rather, nothing is stated concerning the connections between possibly corresponding interneurons and motoneurons of the biological system. The functional separation between Swing-net and Stance-net does only concern the sensory input, whereas both share the motor output and may also share the corresponding interneurons. This is illustrated in Fig. 5 in more detail. The network corresponding to Swing-net is depicted on the left hand side, that corresponding to the Stance-net on the right hand side. This figure, as a summary, combines elements which in part have only been simulated in isolation and which will be characterized in the remainder of this section.

The leg controller receives abundant sensory input which in Fig. 5 is depicted by elements shown in black. This input concerns position and velocity of the joint angles, tactile contact at the surface of the leg and/or loading of the leg, but may also include tactile sensors on the body, as well as sensors monitoring distant stimuli (e.g., eyes, antennae or acoustic sensors).

Let us begin to describe the features of Swing-net of which different versions have been developed. Common to all versions—three will be sketched below—is that Swing-net receives sensory information from the leg joints (position, velocity) as well as information concerning the AEP (provided by a Target-net, see below) and produces signals given to the motor system. These output signals are used to produce a swing trajectory (for simulation of body elements see below). Depending on the specific version, Swing-net can produce searching movements (Dürr 2001; Bläsing 2006) and control avoidance reflexes (e.g., Schumm and Cruse 2006; Dürr et al. 2004)

Swing-net 1 (for details see Dürr et al. 2004) comprises a simple feedforward network (6 inputs, 3 outputs, one for each joint) with a small number of weights. Basically, it consists of three negative feedback controllers, one for each joint. The joint values corresponding to the AEP, as the target position, form the reference values. The $\upbeta$-controller receives crosstalk influence from the $\upalpha$-joint. This connection is responsible for the lifting of the leg during the forward directed movement of the $\upalpha$-joint. A disadvantage of Swing-net 1 is that it cannot directly be used for backward walking. This is different with Swing-net 2, where the lifting of the leg in the $\upbeta$-joint is not produced by coupling it to the $\upalpha$-joint, but by an antagonistic element within the $\upbeta$-controller. This element causes the leg to move upwards first, before it is moved down again. In Swing-net 3 the $\upbeta$-controller is simplified by exploiting the antagonistic architecture of the sensorimotor system as found in the biological system (Schumm and Cruse 2006, their Fig. 7) leading to the structure schematically depicted in Fig. 5. The lifting of the leg is now produced by an inhibitory rebound effect due to mutual inhibition between the neurons driving motor output, in this case, following the biological nomenclature, the Levator-Depressor system. In Fig. 5, the neural structures corresponding to the circuit given by Schumm and Cruse (2006, Fig. 7) are symbolized by two units (depicted in blue) connected by mutual inhibition (as the robot Hector has no antagonistic motor systems, we implemented Swing-net 3 by introduction of a high-pass filter (HPF) into the $\upbeta$-controller only showing the same functionality when walking on a flat surface). Both Swing-net 2 and Swing-net 3 can be used for forward and backward walking by simply using different AEP and PEP memories (being represented by the boxes Target-net in Figs. 4, 5).

The different avoidance reflexes that are excitable during swing have been implemented in Walknet as a simple expansion of Swing-net (Cruse et al. 1998, in Fig. 5 only the Levator reflex is sketched, for details see Schumm and Cruse 2006). More recent versions of Walknet allow for continuation of the swing movement by searching movements when the agent is stepping into a hole (not depicted in Fig. 5, but see Dürr 2001; Bläsing 2006).

For Stance-net, there are several versions, too, but they do not represent possible alternatives as is the case for the three Swing-nets, but address different, functionally separable aspects of one controller which have been simulated only in isolation. Figure 5 shows the complete controller. A general feature is that during stance movement (and during standing still) the body has to maintain a given distance to the ground. This is done by influencing the $\upbeta$-controller by a simple (negative feedback) height controller based on sensory information concerning the leg joint positions, or based on a nonlinear, feedforward network (height controller, Fig. 5) being trained on the basis of biological data (Cruse et al. 1998). This system can be described by a (nonlinear) proportional controller qualitatively characterized by each leg representing a soft spring element acting parallel to the dorso-ventral axis.

To control the movement/force required to propel the body, the simplest solution is the introduction of three negative feedback controllers using the PEP angles as reference values. Such a system is simple to construct but has the disadvantage that, due to the mechanical coupling between the legs, unwanted forces may act across the body requiring unnecessary large torques (Lévy (2009) proposed a possible way to cope with this problem, see below, torque minimization). As an alternative solution the idea of using positive velocity feedback, dating back to the seminal studies of Bässler (1976, 1983), has been applied to Walknet by Cruse et al. (1996), Kindermann (2002) using kinematic and dynamic simulation and by Schmitz et al. (2008) using a physical robot. While during the stance movement all joints connected through body and environment have to be coordinated, the basic idea of positive velocity feedback is that this coordination does not result from explicit coordination and computation. Instead, the influences between all these joints are mediated directly through the body and each joint is locally only trying to continue the externally applied movements on the level of joint velocities. To this end, $\upalpha$-joint and $\upgamma$-joint are subject to positive velocity feedback, whereas the $\upbeta$-joint is controlled by negative position feedback. Following this control architecture, the walker is able to adapt to arbitrary shapes of the substrate, while all 18 joints are governed by local controllers, coupled via the body and the substrate.

As a completely different solution, which is, however, functionally quite similar to positive velocity feedback, a kinematic body model has been introduced to control stance movement (Schilling et al. 2012; Schilling 2011). Such an internal body model forming a neural structure is able to represent any geometrically possible body position, including legs. When used by Walknet to control stance movements, the whole system is functionally similar to the positive velocity feedback approach as it can adapt to any surface structure and allows for easy control of curve walking (Schilling et al. 2012). There are, however, no hints that such an internal body model is used by insects.

For controlling standing still, of course, the above-mentioned simple negative feedback controller could be used, if the reference values for the desired leg positions were given. However, to comply with biological data (see below, Sect. 4), a somewhat more complex circuit has been developed (schematically depicted in Fig. 5) that does not require an explicit specification of the desired leg positions as will be explained in the following.

During standing, as studied by Cruse et al. (2004), all three joints of a leg on the ground appear to be controlled by a specific Integral controller (Fig. 5, $\sum$(1), sensory feedback from $\upalpha$, $\upbeta$, or $\upgamma$, respectively), i.e., a negative feedback system that is able to maintain at zero the error between actual position and desired position (or reference position, given by $\sum$(2) in Fig. 5). For each joint, the integrator required for the Integral controller is given by the box $\sum$(1). This element integrates the controller error, i.e., the difference between the current angle ($\upalpha , \upbeta$ or $\upgamma )$ and the reference value. As shown in Fig. 5, the $\upbeta$-controller in addition depends on input from the height net to control body clearance. Different from a traditional integral controller, under specific conditions (see below), the reference value as such can gradually adapt to a value that is similar or even identical to that of the actual value. This property is due to the positive feedback loop adding the error signal to the integrator represented by $\sum$(2) in Fig. 5. Due to this additional feedback loop, the reference will, after sufficient time, correspond to the actual value. The complete system may therefore be able to show properties of a proportional controller or even a differential controller. The latter properties are approached the more, the stiffer the substrate (Cruse et al. 2004, not depicted in Fig. 5). In this way, the reference value adapts to the current position. Therefore, an explicit specification of the reference values is not necessary. This circuit has been simulated and tested in isolation for one and two joints by Schneider et al. (2007).

In Fig. 5, conceptually, a way is shown how this circuit, used for standing, could be combined with the positive velocity feedback solution explained above being applied for walking (Schneider et al. 2008; Schmitz et al. 2008). Recall that the reference values for the subsequent integral controllers are represented by the boxes $\sum$(2). To obtain an overall system showing positive velocity feedback, the joint controllers of the $\upalpha$-joint and of the $\upgamma$-joint receive additional input representing the angular velocity of the respective joint (see input $\dot{\alpha }, \dot{\gamma }$ in Fig. 5). The $\upbeta$-controller does not receive this input because it is responsible for height control. This hypothesis could explain the—at first glance contradictory—result that legs during stance are also subject to negative feedback (Bartling and Schmitz 2000).

*PEP:* In forward walking, as mentioned above, the posterior extreme position PEP, i.e., the transition from stance to swing, is regarded as the most important parameter at which interleg coordination influences the quasi-cyclic movement of a leg (Cruse 1990). In the free walking animal, PEP should not be considered as a given point in the 3D space, as the distance between body and tarsus may vary depending on the geometry of the current substrate. Measurements in the forward walking animal have shown (Burkamp 1996) that the point where the stance-swing transitions occur is better approximated by a section of an about spherical surface which can be represented by a three-layered feedforward perceptron as indicated by the box PEP in Fig. 5. This “PEP net” has been introduced first to the Walknet version shown in (Cruse et al. 1998). To simplify and speed up the simulation, PEP is, however, usually defined only by the x-coordinate (i.e., parallel to the long axis of the body), a suitable simplification as long as relatively even surfaces are used. The PEP value can be influenced by the coordination rules (see below) and by load. The latter has been introduced in Walknet by Schilling et al. (2007), whereby increased load shifts the PEP to the rear.

AEP: The transition from swing to stance is triggered by the swinging leg receiving ground contact. In earlier Walknet versions, ground contact of a leg was represented by a Boolean variable. In the version of Schilling et al. (2007), instead of ground contact, an analog value representing load has been introduced which, when reaching a given value, activates the stance motivation unit which in turn suppresses the swing motivation unit (Fig. 5, for explanation of motivation units see below). In earlier versions, the sensory input to these units, now termed motivation units, was termed “selector net” (e.g., Dürr et al. 2004).

### Coordination rules

As was mentioned earlier, legs may be coupled via mechanical and/or neuronal influences. Several rules that describe the coordination between legs were derived from behavioral experiments on forward walking stick insects (Cruse 1990) and have been implemented by Dean 1991a, b, 1992a, b; Calvitti and Beer 2000). In Fig. 3, the rules are numbered 1–6. Coordination rules 1–3 influence the length of the stance movement by influencing the transition from stance to swing movement, i.e., they change the PEP value. Rule 4 is represented by a specific version of the Target-net (Dean 1990) that transmits the current position of the anterior leg which is used as the swing target by the receiving leg. In other words, rule 4 influences the AEP. As the critical experiments have been performed with animals where the mechanical coupling between legs has been excluded (Cruse 1979b; Cruse and Epstein 1982; Cruse and Schwarze 1988; Cruse and Knauth 1989), a (yet unknown) neuronal basis has to be postulated for rule 1–4.

Based on studies of Graham (1972, his Figs. 7, 8) using free walking insects, the parameters describing rule 3 depend on walking velocity (Dean 1991b). This dependence is different for rule 3 acting between ipsilateral legs (from front to rear in forward walking) and acting between contralateral leg pairs. Together, these connections contribute to the formation of a recurrent network. The following shows a verbal description followed by a pseudocode formulation of how rules 1–3 are implemented in the actual version of Walknet for forward walking (all distances are given in m, distance between default AEP and default PEP is 0.3 m).

*Rule 1* inhibits the beginning of swing of the receiver leg as long as the sender leg is in swing plus a given velocity-dependent delay (delay_1b) after swing is finished. This is done by shifting the PEP to the rear and may inhibit the start of a swing. Rule 1 operates between ipsilateral legs from back to front.

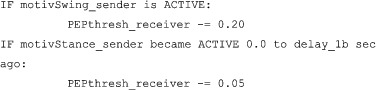



The delay is velocity dependent:

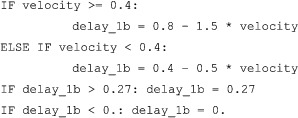



*Rule 2* shifts the PEP forward after the sender leg has started stance plus a given delay (270 ms). This influence is active for 50 ms. Rule 2 may elicit the start of a swing when the receiver leg has moved far enough to the rear. Rule 2 operates between ipsilateral legs from back to front and between contralateral legs.

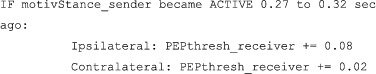



*Rule 3* shifts the PEP of the receiver forward when the sender leg has reached a given position during stance, thereby possibly eliciting a swing of the receiver leg. The critical position of the sender leg depends on velocity and is different for influences between ipsilateral legs and between contralateral legs. This influence is active during a given position window of the sender leg. Rule 3 operates between ipsilateral legs from front to back and between contralateral leg pairs in both directions.

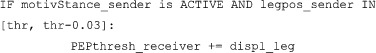



The threshold thr is velocity dependent:

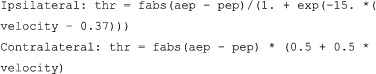



The displacement of the PEP depends on the direction and the leg of the influence:

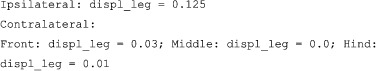



Of course, in the free walking animal, mechanical effects may contribute, too, which may particularly be true for the velocity dependent part.

When load of a leg is increased, there is, as has been mentioned above (3.1), a local effect that PEP is shifted to the rear (or to the front after deloading the leg) and, at the same time, there is a coordination effect in that neighboring legs develop higher forces. The latter has been termed rule 5. Schilling et al. (2007) implemented both effects in a way that the load signal used by each individual leg controller to support the decision between stance and swing is also given to the controllers of the neighboring legs. If above threshold, these signals are simply added to the local signal recorded by the leg (not depicted in Fig. 5).

Rule 6 will be addressed in Sect. 4 as it has not been implemented in Walknet up to now.

### Motivation units

Walknet in the version described earlier (Dürr et al. 2004) represents a network that is only able to control forward walking. To allow the system to select autonomously between different behaviors, Walknet has been expanded by introduction of a network consisting of so-called motivation units, in Figs. 4 and 5 marked in red (Schilling et al. 2007; Schilling and Cruse 2008, for details see Schilling et al. submitted). Examples for such different behaviors concern the selection between standing and walking, between forward and backward walking (Schilling et al. submitted; Cruse and Schilling 2013) or, as a future extension, a Mantis-like four legged walking where the front legs could be used as grippers instead of legs. In the latter case, in addition to the procedures for swing and stance, for the front legs procedures have to be introduced that allow for the control of gripper movements.

Such an architecture has also been applied for a simulation system called Navinet (Cruse and Wehner 2011; Hoinville et al. 2012) that controls insect navigation, where the animals are able to select between different food sources learned, between traveling to a food source or back to home, or to attend to a learned landmark depending on the actual context.^2^

Motivation units are used here to represent the strength of a motivation to perform the corresponding behavior. Following Maes (1991) and Hassenstein (1983), both inspired by K. Lorenz, the ability to perform a behavior depends on a cooperative influence of the relevant sensory stimuli and the strength of its motivation. Common examples for motivational states are aggression controlling fight, or fear controlling flight. But depending on the granularity of how behavioral elements are defined, motivational states may also concern “microbehaviors.” Examples relevant in the case of Walknet are swing or stance of a leg.

Motivation units are applied in two ways. First, motivation units can determine the output of a procedure, which is, in the simplest case, done by multiplication (as a more neuronal version based on antagonistic structures inhibitory connections may be applied). To this end, each procedural element is equipped with a motivation unit. Therefore, not only Swing-net and Stance-net as in Schilling et al. (2007), but also all Target-nets (Schilling and Cruse 2008) as well as the leg coordination channels are equipped with an own motivation unit.

Second, motivation units can also be used to influence other motivation units via excitatory or inhibitory connections. As illustrated in Fig. 4, motivation units controlling the procedures of one leg are connected to a “leg” unit, and all leg units are in turn connected to a unit “walk.” Furthermore, there are units for forward walking (“fw”) and backward walking (“bw”). This at first sight hierarchical structure is, however, not forming a simple, tree-like arborisation. As indicated by the bi-directional connections, motivation units form a recurrent neural network coupled by positive (arrowheads) and negative (T-shaped connections) influences. This structure may therefore be better described as “heterarchical.” For example, some of these motivation units are coupled by local winner-take-all (WTA) connections. This is true for the Swing-net and Stance-net of one leg, as well as for the different Target-nets controlling the same leg. Only one of the units belonging to such a WTA net can be active at any time. For example, only one of the available Target-nets can be selected, depending on which motivation unit, “fw” or “bw,” is active. Excitatory connections between motivation units allow for building coalitions, or clusters. As depicted in Fig. 4, there are different overlapping ensembles possible. For example, all “leg” units and the unit “walk” are activated during backward walking and during forward walking, but only one of the two units termed “forward” and “backward” and only one of the motivation units of the Target-nets are active in either case thus allowing for the selection of the appropriate Target-net.

The organization of the motivation unit network allows for competition on different levels. For example, on the leg level the competition selects swing and stance movements while on a more global level, the walking direction (forward/backward) or the overall behavior like walking or standing still can be selected.

In this way, this network produces various stable attractor states or “internal states.” Such internal states not only allow for selection of behavioral elements, but also provide a context according to which specific sensory inputs are attended or not. For instance, as a lower-level example, depending on whether a leg is in swing state or in stance state, a given sensory input can be treated differently: stimulation of a specific sense organ (Fig. 5) leads to a Levator reflex when in swing, but not during stance. On a higher level, for instance forward walking or backward walking, different sensory input is used to determine AEP and PEP. As a more interesting example, in the above-mentioned Navinet, visual input characterizing a known landmark is only attended when belonging to the landmarks leading to the actually addressed food source.

As is the case for the motivation units of Swing-net and Stance-net, also the “higher-level” motivation units may receive direct or indirect input from sensory units that influence the activation of this motivation unit (not depicted in Fig. 4).

The function of motivation units have, for Swing-net and Stance-net, already been used in earlier versions of Walknet in the form of the output units of the so-called selector net (e.g., Dürr et al. 2004). Mutual inhibition has not been applied in these earlier versions because training the motivation unit network lead to another distribution of the weights (Cruse et al. 1993a, 1995a). Mutual inhibition has been introduced by Schilling et al. (2007). Motivation units for forward and backward walking have been applied in the so-called “distributor net” (Schilling and Cruse 2008, their Fig. 9). To switch between forward walking and backward walking, Ayers and Davis (1977), and more recently Tóth et al. (2012), have proposed very similar circuits. Ayers and Davis (1977) named their control units command neurons.

### Simulation of the body and the environment

Walknet versions differ with respect to the degree to what extent the simulation of the environment, in particular the body, has been realized. Earlier versions used a simple kinematic simulation of the body. Later versions used different ways of how to dynamically model the body. Finally, Walknet has been tested on various robot platforms (Espenschied et al. 1993; Ferrell 1995; Flannigan et al. 1998), including the TUM walker (e.g., Pfeiffer et al. (1995), Tarry II (Frik et al. 1999), and Tarry IIB (e.g., Schmitz et al. 2008). The currently developed hexapod robot Hector contains serial elastic elements (Paskarbeit et al. 2010; Schneider et al. 2011) to provide a first approximation to the properties of biological actuators.

Simulation of coupling via the substrate was, in an early kinematic version of Walknet, simplified by assuming all legs in stance to adopt the same velocity (Müller-Wilm et al. 1992; Cruse et al. 1993a, 1995a, b). In later, still kinematic simulations, based on controlling the stance movement via positive velocity feedback, these mechanical contributions are implemented explicitly (Cruse et al. 1996, 1998; Kindermann 2002). Mechanical coupling between legs is also addressed in the more recent dynamical simulations (Schilling et al. 2007; Schmitz et al. 2008; Schneider et al. 2011) and, of course, by the hardware simulations, i.e., the robots mentioned above.

Although eventually criticized as being too simple, kinematic simulations might not be a too bad approximation for a walking stick insect (different to a robot), because body masses, in particular those of the legs, are small and friction in the joints is high (e.g., Hooper et al. 2009). Muscles have to date not explicitly been implemented in Walknet. Instead this function is roughly approximated by the torque generators used in the dynamic simulation approaches and the application to physical robots. Recently, Annunziata and Schneider (2012) have developed a muscle simulator for the motors that are actually being implemented in the robot Hector which will be controlled by Walknet.

## Biological results and their simulation

What is known from biology concerning the control of six-legged walking and which aspects are covered by Walknet? In this section, we will briefly review the most important results and conclusions taken from behavioral studies of several species of stick insects (*Carausius morosus, Areaton asperrimus*). We will deal with questions concerning different aspects of, first, coordination of the different procedures via motivation units, then coordination between legs and later the control of the individual leg.

Let us begin with some general aspects. Properties of Walknet have been tested in several versions, all sharing the following properties: Using a realistic leg morphology (Fig. 1), they show gait patterns as observed in stick insects including continuous gait transitions from very slow wave gait to slow tetrapod gait to fast tripod, depending only on the velocity command (see Schilling et al. (submitted) for an analysis of the effect of different velocities on the emerging gaits). All the Walknet versions deal with various disturbances applied during walking in a similar way as found in the insects during swing and stance (see below). They can deal with obstacles of limited height (systematically investigated by Kindermann 2002). All Walknet versions show sign reversal in joint movement during swing and/or during stance movement as observed in the insects (e.g., Cruse and Bartling 1995), which is particularly interesting with respect to the $\upgamma$-joint during stance.

### Motivation units

Neuronal elements that gate the activation of lower-level procedures are known or postulated since long.^3^ With respect to insect walking direct or indirect evidence for such elements is given for higher-level functions as are decisions on walking direction (right-left), or start and stop (see Strauss 2002). Neurophysiological grounding for the unit “walk” is given by Büschges (1998): when walking is started, the membrane potential level in a number of relevant motoneurons is tonically increased. The motivation units influencing the coordination rules are motivated by Dürr (2005); Ebeling and Dürr (2006), who showed that coordination influences can be modulated (e.g., during curve walking). Results of Cruse et al. (1998) support the introduction of a motivation unit for the procedure controlling swing movement. This is also the case for motivation units of Target-nets due to the assumption that targeting, i.e., rule 4, seems to be switched off during curve walking (Dürr 2005; Dürr and Ebeling 2005; Rosano and Webb 2007; Gruhn et al. 2009). Mutually inhibitory neurons functionally corresponding to the motivation units that are assumed to control forward and backward walking as depicted in Fig. 4 have been introduced in the model of Tóth et al. (2012). Interestingly, introduction of these units is based on neurobiological insights obtained (Büschges 1995; Wildmann et al. 2002). A switch between states on the neuronal level has been postulated by Bässler (1983) when the animal changes from standing (passive) mode to walking (active) mode.

### Leg coordination

The basic assumption, that the architecture of the walking controller consists of individual modules (Fig. 3) each controlling the movement of one leg, is well supported by many experiments (behavioral observations date back to Buddenbrock 1921; Bässler 1983; Wendler 1964, for neurobiological results see a review by Bässler and Büschges 1998). These modules are influenced by central commands controlling start, stop, velocity and direction of walking. Results on where this information resides within the insect brain are given by Strauss (2002) and Neuser et al. (2008) as studied in detail in the fruitfly Drosophila.

Coordination rules 1–6 have been derived from behavioral studies. Such studies suffer from a general dilemma: On the one hand it is necessary to study the complete (i.e., intact and freely behaving) animal to avoid artifacts possibly produced by restraining the animal (this problem is of course particularly true for neurophysiological studies with restrained animals). The holistic behavioral approach, on the other hand, does not allow a clear attribution of the observed phenomena to specific local mechanisms, for example what effects result from neuronal mechanisms and what from mechanical properties.

Rules 1–3 have been studied on slippery surfaces to exclude effects of mechanical coupling (Cruse and Epstein 1982; Cruse and Schwarze 1988; Cruse and Knauth 1989), where leg movements have been disturbed during stance or swing by controlled mechanical stimuli (see below). Comparable behavioral effects have been observed in less artificial situations (walking on a treadwheel, i.e., with mechanical coupling between legs (Bässler et al. 1987; Dean and Wendler 1982, 1983; Foth and Bässler 1985; Graham 1972) which suggests that no essential artifacts are produced with respect to leg coordination when walking on the slippery surface. As the simulations provide good descriptions of the walking patterns observed in free walking insects, these rules represent a sufficient hypothesis concerning the existence of underlying neuronal coupling mechanisms. These rules, however, contain no information as to how those couplings are realized on the neuro-muscular level and provide a concise description of a number of behavioral results, results that should be replicated by alternative modeling approaches.

Studying the effects underlying rules 4–6 was possible by controlled experiments, allowing for more direct evidence. Rule 5 addresses the distribution of forces between legs and the coactivation of neighboring legs in order to distribute load (Cruse 1985a; Schmitz 1993; Schmitz et al. 1999). In earlier Walknet versions, this rule has not been explicitly implemented as load sensors have not yet been introduced. An explicit simulation of load influences is, however, given by Schilling et al. (2007), as addressed below (Sect. 4.3).

Not simulated at all is the coordination rule 6, also termed treading-on-tarsus (TOT) reflex (Graham 1979). If, at the end of the swing movement of, for example, the middle leg, the tarsus of the ipsilateral front leg has received a tactile stimulus, the ipsilateral middle leg performs a short back step. This situation happens when the middle leg has accidentally stepped onto the tarsus of its anterior neighbor. In this way, the TOT reflex leads the leg away from the anterior leg and prevents stumbling. Interestingly, in backward walking animals the TOT reflex is active, too, but leads to a behaviorally/physiologically unreasonable reaction (Schmitz and Hassfeld 1989). In this situation still the tarsus of the front leg has to be stimulated when the ipsilateral middle leg is at its end of the swing. This leads to a forward step of the middle leg. However, in backward walking the middle leg is now near its posterior extreme position, far away from the front leg (which is now near its anterior extreme position). It is not possible to elicit TOT reflexes in the backward walking middle leg by stimulation of the hind leg tarsus. Neurophysiological studies (Schmitz and Hassfeld 1989) revealed that the reflex in backward walking animals affects the functional swing and stance muscles in qualitatively the same manner as in forward walking. In other words, in forward walking, the stimulation of the front leg tarsus leads to an inhibition of a functional swing muscle, the Protractor, and an activation of the Retractor. In contrast, in backward walking the same stimulus leads to an inhibition of the functional swing muscle, which is, however, now the Retractor, and an activation of the Protractor. Thus, whether walking forward or backward, a brief swing is elicited opposite to the direction of the actually executed swing movement, a sensible behavior in forward walking, but not in backward walking. This is a strong indication for a hierarchical organization of the leg step generator. The motoneurons of the swing muscle are inhibited and those of the stance muscle are excited. This shows that the stimulus information from the front leg is gated by the actual state of the higher level of the step generator which determines swing and stance. The lower level of the step generator then decides, depending on the walking direction, forward or backward, which motoneuron pools have to be addressed. How such a circuit could be implemented in our framework is depicted in Fig. 4, dotted lines. To this end, mutual inhibition between both Target-net motivation units is required.

In neurophysiological studies, Borgmann et al. (2007, 2009) found influences between neighboring leg controllers that could be interpreted as to correspond to rule 5. Indirect evidence could be provided by Brunn and Dean (1994) who found three neurons that are able to project information concerning the three joint angles of one leg to the adjacent posterior ganglion, which might therefore be responsible for the information transfer required for rule 4.

A coupling influence corresponding to rule 1 is active in backward walking animals and shows a functionally analogous behavior as during forward walking although the target motoneuron pools are inverted; in so far these reflexes change sign. The same is in principle true for rule 3, although strength and phase appear to be somewhat different (Düsterhus and Schmitz 2009; Schmitz and Düsterhus 2009).

Coordination influences may vary in strength depending on the actual context as studied for curve walking by Dürr (2005) and Dürr and Ebeling (2005), which, in Walknet, would be possible by modulating rules’ strength using additional motivation units (as depicted in Fig. 4).

*Gait types:* The neuronal and mechanical coupling influences lead to spatio-temporal leg patterns generally described as tripod gait, tetrapod gait or wave gait (e.g., Grabowska et al. 2012). These terms must not, however, be understood as characterizing separate gait types as are trot or gallop observed in mammals, but rather describe a continuum (Graham 1972, 1985). The so-called tetrapod gait (Fig. 2) gradually changes into a tripod gait when walking speed of the animal is increased. Correspondingly, wave gait gradually changes into tetrapod gait. This effect is due to an about constant delay (for its velocity dependence see Graham 1972, his Fig. 7, for simulation results see below) between swing of hind leg and (ipsilateral) middle leg as well as between swing of middle leg and (ipsilateral) front leg and continuously varying stance duration depending on the parameter walking velocity. This continuum is not a specific property of stick insects but has been also observed in other insects in which slow walking has been studied (for cockroach see Bender et al. 2011, for Drosophila see Wosnitza et al. 2013). Therefore, tripod pattern should not be considered as a different type of gait but rather the upper end of a continuum. Even more, idealized patterns like tripod or tetrapod can only be observed when the animal is walking on a homogeneous surface and at a specific constant velocity. Any disturbance will lead to a more or less obvious deviation from the ideal pattern. In other words, the properties of the environment play a crucial role in shaping the actual pattern. Therefore, as a better description the term “free gait” should be applied, which has been used by different authors to classify gait types (Dürr 2005). This term implies that the gaits observed are not rigidly implemented into the neural structures (different to the assumption of Steingrube et al. 2010) but can be characterized as emergent properties (corresponding results have been found for crayfish walking, Cruse and Müller 1986). As a specific example for further influences on the leg pattern, Graham (1985, his Fig. 17) has shown that the tripod pattern also changes into a tetrapod pattern when horizontal load is increased. This is the case, for example when insects are walking on a mercury surface (Graham and Cruse 1981), swing duration is not anymore constant, but increases with stepping period, i.e., increases with decreasing walking velocity. The footfall pattern resembles a tripod, also for lower velocities, but with keeping the ratio between swing duration and stance duration about constant. This velocity dependence of swing duration has not been simulated by Walknet. However, in a precursor model (Cruse 1983), it has been shown how this might be possible.

**Fig. 6:**
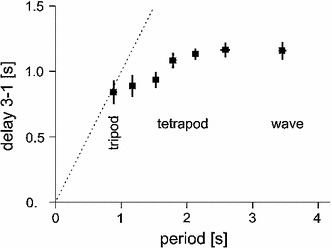
Delay 3-1, time from the beginning of a swing in the hindleg to the beginning of the swing of the ipsilateral front leg (see Fig. 2) versus period, mean $\pm$ SD (compare Graham 1972, his Fig. 7). The regions characterizing behaviorally defined stepping patterns are marked

As demonstrated by the Walknet simulations, coordinating rules 1–4 and the mechanical coupling are sufficient to explain properties described as tripod, tetrapod or wave gait patterns including smooth transitions. Figure 6 shows the behavior of Walknet in the same format as applied by Graham (1972, his Fig. 7). Both results are in good agreement apart from the fact that the temporal scales differ by about a factor of two. These observations are in contrast to suggestions of Daun-Gruhn and Büschges (2011) who appear to assume that dynamical models based on differential equations are required to simulate smooth transitions between gaits. Furthermore, using these coordination rules, Walknet shows regular oscillations of instantaneous walking speed (Kindermann 2002, his Figs. 8, 9) during a complete step as observed by Graham (1972), stable reactions to various disturbances during stance or swing, converging to a stable walking pattern when starting from arbitrary leg configurations, climbing over obstacles, dealing with leg amputations and recovery from stumbling (all effects studied by Kindermann 2002) as well as, with some minor expansions (Bläsing 2006), climbing over gaps as wide as the length of the own body (about twice the normal step length).

*Curve walking:* By choosing different velocities for right and left legs, curve walking is possible in principle (e.g., Kindermann 2002) and has been used in older versions of Walknet, but Walknet does not show the ability to perform side steps as observed in stick insects (Dürr 2005; Dürr and Ebeling 2005; Rosano and Webb 2007; Gruhn et al. 2009; Cruse et al. 2009, for crayfish see Cruse and Silva Saavedra 1996). The mechanical influences on positioning of leg extreme positions during curve walking has been studied by Kindermann (2002, his Figs. 12, 13). Interestingly, the extension to Walknet proposed by Rosano and Webb (2007) is able to quite well replicate the curve walking behavior of stick insects. This is also true for the Walknet version using the internal body model (Schilling et al. 2012), both results indicating the importance of body kinematics.

*Leg amputation:* Walknet is able to overcome the loss of up to two legs as has been found in insects (for biological results see Buddenbrock 1921; Wendler 1964; Graham 1977). The present coordination rules together with the coupling through the environment have shown to be sufficient to coordinate the remaining leg’s behavior for most configurations (Kindermann 2002; Schilling et al. 2007). Only in the case of the loss of a middle leg, the introduction of a new coordinating influence connecting hind and front leg becomes necessary. This influence depends on the load acting on the middle leg as already hypothesized by Wendler (1968) and by Graham (1977). Recent results on leg amputation provided by Grabowska et al. (2012) have not yet been simulated by Walknet, but as these results very much agree with those of earlier studies, they might well be replicated by a Walknet controlled hexapod, too.

*Gliding coordination:*von Holst (1939, 1943) introduced the term of relative (or gliding) coordination to be distinguished from absolute coordination. In the latter case, two coupled oscillators with somewhat different eigenfrequencies differ only by a fixed phase value due to a strong coupling, whereas in gliding coordination any phase difference is possible, but particular phase values occur more often than others. Gliding coordination (also called “magnet effect”) has been observed in free walking lobsters (Chasserat and Clarac 1980), for example, and has been shown in stick insects between front leg and hind leg after loss of the middle leg (Wendler 1964, his Fig. 7). In a simulation of crayfish walking based on the same principles as Walknet, but using coordination influences found for crayfish, gliding coordination between contralateral legs has shown to be possible, when the coordination influences are small enough (Müller and Cruse 1991).^4^ Note that gliding coordination defined this way is conceptually different from the observation that leg oscillators show different phase values when walking velocity is varied.

### Control of the individual leg

A large number of studies concern the investigation of the system controlling the movement of the individual leg. In the form of a network diagram, Fig. 5, forming a quantitative functional hypothesis, summarizes the most relevant findings that will be treated in the following.

*Standing still and walking:* Basically, two different behavioral modes have to be distinguished, standing still, thereby supporting the body weight, but also resisting to additional external forces applied to the body, and walking. When in walking mode, the leg has to adopt one of two submodes, stance or swing. During stance, the legs support the body and move it in the desired direction (forward, backward, or sideways, e.g., during curve walking). While standing, legs support the body weight, but distribution of load may vary. For example, an individual leg may also be unloaded, keeping its position fixed, but not carrying any body weight (see below, torque minimization).

During standing, all three joints of a leg on the ground have been described as to be controlled by a negative feedback controller with a strong phasic component (Bässler 1983 for Femur-Tibia joint, Schmitz 1986a, b for the Coxa-Trochanter joint, Graham and Wendler 1981; Schmitz and Stein 2000 for the retractor- protractor system), i.e., a negative feedback system that also reacts to the velocity of a disturbance signal. This result is, however, only observed when the leg is moved via a stiff substrate. If the substrate is compliant, on a substrate of medium stiffness the controller shows properties of a proportional position controller, whereas on a substrate compliant enough the leg is able to maintain the error between actual position and desired position (reference position) constant, i.e., the controller behaves as an Integral controller (Cruse et al. 2004). This is replicated by the controller of a standing leg as depicted in Fig. 5. Each joint controller shows properties of an Integral controller (Fig. 5, $\sum$(1)), which, however, “intelligently gives up” this behavior when it is ineffective as is the case when standing on a stiff substrate. As explained earlier, this circuit has been simulated and tested by Schneider et al. (2007) in isolation for a single joint and a planar leg consisting of two joints.

Experimental results from walking animals (Bartling and Schmitz 2000) where the position of the tarsus was, during stance, briefly moved forward, backward, inside or outside, indicate that in a walking animal all three joints are subject to negative feedback (Cruse and Schmitz 1983; Schmitz 1985). Therefore, the basic version of Stance-net consists of a negative feedback controller for each joint where the reference input is given by the PEP angles. On the other hand, further experimental results indicate that at least in the $\upgamma$-joint, positive velocity feedback exists in the walking animal (Bässler 1983, 1976; Schmitz et al. 1995), allowing for the so-called “active reaction” (whereas only negative feedback could be found in the $\upbeta$-joint (J. Schmitz, pers. comm.). Using kinematic simulations, Kindermann (2002) could show that positive velocity feedback in the $\upalpha$-joint and the $\upgamma$-joint, combined with negative position feedback in the $\upbeta$-joint is actually suited to control stable walking of a hexapod. The critical experiment, the test on a robot, has been performed by Schmitz et al. (2008).

How both approaches, positive velocity feedback and the type of negative feedback studied by Schneider et al. (2007) may be combined in the walking animal is indicated in Fig. 5. To this end, the reference value for the controllers of the $\upalpha$-joint and the $\upgamma$-joint are influenced by the corresponding angular velocities ($\dot{\alpha }, \dot{\gamma }$, Schneider et al. 2007) which represents the positive velocity feedback. This combination has, however, not yet been tested.

*Height control:* During both, standing still and walking while in stance mode, all leg joints are subject to a negative feedback system controlling the height (body-ground distance) (Cruse 1976a, b; Cruse et al. 1989, 1993b). This system can be described by a (nonlinear) proportional controller qualitatively characterized by each leg representing a soft spring element acting parallel to the dorso-ventral axis (Fig. 5, height control). The properties of height net were trained off-line using data obtained from biological experiments with standing and walking animals (Cruse et al. 1995a, b). The dynamic properties of this height controller depend on the mode (walking or standing), as the time constants are much smaller during walking compared to standing (Cruse et al. 1989, 1993b; Schmitz 1985). The fast version of this height controller has been implemented in Walknet by Cruse et al. (1995a, b) and at least qualitatively describes the biological findings when walking over uneven surfaces (Kindermann 2002, his Fig. 14). A quantitative simulation was not possible because apart from the latest robot version, Hector, in all earlier versions the thoracic joints were assumed to be fixed, in contrast to the situation found in stick insects. When a stick insect walks over uneven surfaces, the joint allowing for up-down movements between mesothorax and metathorax shows the properties of a linear elastic element (Cruse 1976b). This property could be due to a proportional position controller in this joint and can now easily be implemented in the controller ruling the corresponding joint in Hector (Paskarbeit et al. 2010; Schneider et al. 2011).

*Interruption during stance*: Reactions of the animals to disturbances applied to a leg tip during stance in different directions (front, back, inside, outside) (Bartling and Schmitz 2000; Cruse 1985b) can in principle be simulated by the Stance-net consisting of negative feedback controllers. However, in these experiments the legs counteract the disturbance only briefly and do not appear to return to the reference value during the remainder of the stance movement as one could have expected with a traditional position feedback controller being applied. This observation might be explained by the above-mentioned property of the Integral controller to “intelligently give up,” i.e., a differential, or velocity, feedback controller. However, as mentioned above, there is no quantitative simulation of the complete controller as depicted in Fig. 5, i.e., the combination between Integral controller and positive feedback influence.

Short and long disturbances in vertical direction have been applied to walking animals (Cruse et al. 1993b) and to standing animals (Cruse et al. 1989). As these results have been used to design the height controller, they can be replicated by Walknet.

The observation that PEP can be influenced by loading or unloading the leg itself (for experimental results see Bässler 1977; Cruse 1985c for the stick insect and Pearson (1972) for the cockroach) has been implemented in Walknet by Schilling et al. (2007) through introduction of analog motivation units replacing the earlier Boolean units (see above, 3.1, 3.2).

*Interruption during swing:* A number of disturbances have been applied to study the properties of the controller responsible for producing the swing trajectory. Dean (1984) could show that swing movement is under the control of a negative feedback system. Further studies concern the experimental prolongation of swing, from short prolongations up to those of more than the duration of a stance movement (Schumm and Cruse 2006; Cruse and Epstein 1982; Dean and Wendler 1982). Schumm and Cruse (2006) studied swing trajectories when starting swing at different positions in the leg’s workspace. As exactly these data are used to construct Swing-net 3, they can be replicated by Walknet, too (Schumm and Cruse 2006). Swing-net 3 integrates the properties of the earlier versions. Studies of Schumm and Cruse (2006)—walking on small or broad substrate, and Diederich et al. (2002)—walking horizontally along an inclined surface—have shown that swing movements appear not to reach an absolute dorsal extreme position, but to lift the tarsus by a given amplitude. This property and the fact that swing trajectories show different spatial orientations depending on the horizontal distance of the tarsi during stance (walking on small or wide substrate, respectively) can be replicated by Swing-net 3, too (Schumm and Cruse 2006). The variation of the spatial position of swing trajectory found in these studies can not, however, be simulated by the reduced version of Swing-net 3, where the high-pass effect is only given to the $\upbeta$-controller.

*Levator reflex:* When a leg during swing perceives a tactile stimulus, it may show one of several avoidance reflexes depending on the site of the stimulus (Ebeling and Dürr 2006; Schumm and Cruse 2006). A mechanical contact at the front side elicits the so-called Levator reflex, where the leg is briefly retracted and lifted, before the swing movement is resumed again. Activation of this reflex, however, depends on the internal state (“motivational state”) of the leg controller. During stance, no levator reflex is started, during swing, depending on the distance to the anterior leg, a tactile stimulus may lead to a grasping reflex instead of an avoidance reflex (Cruse et al. 1998). The different avoidance reflexes that are excitable during swing have been implemented in Walknet as a simple expansion of Swing-net (Cruse et al. 1998, in Fig. 5 only the Levator reflex is depicted). The motivational change between Levator reflex and the grasping reflex have been simulated, but not yet integrated in Walknet (Cruse et al. 1998).

*Searching behavior: *When legs are stepping into a hole and do not perceive ground contact, legs show a quasi-rhythmic oscillating movement called searching behavior (Dürr 2001, for qualitative observations in cockroaches, see Pearson and Franklin 1984). More recent versions of Walknet allow for searching movements when the agent is stepping into a hole. One version exploits properties of the feedback controller forming the Swing-net where the network parameters are chosen appropriately (Dürr 2001). The other version uses an internal oscillator as part of Swing-net to produce searching movements of two different frequencies as observed in stick insects (*Aretaon asperrimus*) when trying to climb over large gaps (Bläsing 2006). Searching behavior has also been studied in a somewhat different paradigm where the animal was not walking, but five legs being restrained so that only one leg, usually the front leg, could be moved (Bässler et al. 1991; Karg et al. 1991). Only the two distal joints of this leg were free to move and search was not elicited by missing ground contact, but by tactile stimulation of the leg. This behavior has not yet simulated with Walknet, but might well correspond to a grasping reflex mentioned above, followed by a searching behavior as studied here. In related studies Matheson and Dürr (2003) describe leg movements in locust where the hind leg performs searching movements targeted to different positions on the wing defined by a tactile stimulus.

*Short steps:* When walking over irregular ground, for example approaching a gap or an obstacle, stick insects perform so-called short steps either when starting swing at the PEP or after having reached the anterior extreme position (AEP, Fig. 1) (Bläsing and Cruse 2004a, b; Theunissen et al. 2012). The exact stimuli required to elicit this behavior are unknown. As a simple assumption concerning simulation of the former type, Bläsing (2006) introduced the mechanism that the AEP of a leg is moved to the rear if walking velocity is decreased below a given threshold and when two contralateral legs of the same body segment perform simultaneous swing movements. Walknet being equipped with the ability to perform short steps and the above mentioned searching behavior provides an impressive simulation of stick insect behavior crossing very large gaps (Bläsing 2006) as observed in the animals (Bläsing and Cruse 2004a, b).

*Standing leg of the walking insect:* Stick insects can walk on a treadwheel with five legs while the sixth leg is placed on a platform at a position fixed relative to the body as studied by Bässler (1979), Cruse and Saxler (1980), Cruse and Schmitz (1983), Schmitz (1985) (for similar results concerning the rock lobster see Clarac and Cruse 1982). Even though this situation appears quite unphysiological in the sense that such a situation may not happen in nature, this experimental setting provides relevant information when constructing hypotheses concerning the structure of the walking controller. The “standing leg of the walking animal” develops rhythmically varying forces that depend on the movement of the other legs. Although behaviorally probably not relevant, a controller that is designed to cover the properties of the biological system should be able to describe these results. Using a Walknet version applying what is now called motivation units and introducing connections corresponding to rule 5 Schilling et al. (2007) could indeed explain these results qualitatively.

*Torque minimization*: An interesting property, not depicted in the schema of Fig. 5 and not implemented in Walknet, refers to the fact that a system with six legs, each equipped with three controllable joints, forms a system with extra degrees of freedom. This means that the same body position can in principle be reached by an infinite number of combinations of torques developed in the different (at least 18) joints. Indeed, when excited for example by a tactile stimulus, the legs of a stick insect develop forces in such a way that the ground reaction forces measured at the tarsi increase and are directed toward each other in order to fix the body to the substrate. After the excitation is stopped, the animal relaxes and the torques slowly decrease to an overall minimum. However, in spite of dramatic torque changes, during the complete duration of the experiment, the geometrical position of body and legs is not changed. This is only possible if the 18 torque values are extremely well attuned to one another. Results show that different solutions of approximating an overall minimum of torques are possible and are adopted by the animal (Lévy and Cruse 2008). Simulation studies based on the above-mentioned integral controllers provide a simple, decentralized solution to this problem of dealing with redundant degrees of freedom and finding an energetically cheap solution while maintaining body position (Lévy and Cruse 2008). These results show that animals do not always adopt an energetically minimal solution but can and do exploit the redundancy given by the system. Even when adopting a minimal solution, many different distributions are possible and used. It has been hypothesized that this solution may also be applied during walking (Lévy 2009).

## Related work and open questions

*Representation of the states Swing and Stance:* A family of models, although different in detail, shares the property to operate without an explicit representation of the states Swing and Stance, different to the solution represented by the selector net in earlier versions of Walknet and by application of motivation units in later versions (e.g., Schilling et al. 2007 and Fig. 5). Instead, in these models (Daun-Gruhn 2011; Daun-Gruhn et al. 2012; Daun-Gruhn and Büschges 2011; Ekeberg et al. 2004; von Twickel and Pasemann 2007; von Twickel et al. 2011, 2012) the walking rhythm of the leg is based on the alternating states of one of the joints. These approaches are successful as they allow single-leg stepping, which scales up to hexapod walking, using a small number of neuronal units. But, even compared to earlier versions of Walknet, they only describe a limited range of behaviors. These limitations do not only concern the simulation of a number of specific behaviors observed in walking stick insects like gap-climbing and reaction to various disturbances as mentioned earlier, but also concern straight, undisturbed forward walking. A general drawback of approaches avoiding explicit representation of swing and stance is their property that the motor output—measured as motor neuron activity or as torques produced by the muscles—of the different joints of a leg follows fixed phase relations. Under this assumption, a separate representation of the states Stance and Swing independent of the activation of the individual joints is indeed not necessary. This assumption is, however, generally not in agreement with experimental findings [except for stick insects constrained to walk on a slippery surface (Rosenbaum et al. 2010)]. During stance movement, in free walking animals in the second part of stance movement, retractor torque co-occurs with extensor torque (Cruse 1976a for middle leg torques, Tab. 6, Tab. 7 and for hind leg, Tab. 9, upward walking) whereas, when walking while hanging from a horizontal beam, retraction movement co-occurs with protractor torque in all three legs (Cruse 1976a, Tab 8). Furthermore, when negotiating tight curves, in the inner middle leg, the Protractor may be co-activated with the Flexor during stance (Gruhn et al. 2009, Fig. 3Aiii). In addition, in the hind leg, for example when walking while hanging from a horizontal beam, the Protractor is always active during stance (Cruse 1976a). To our knowledge, there are no data in insects concerning motor activities when walking down a vertical path, but due to the physical situation, it can be assumed that in this situation in the hind leg the Flexor (together with the Retractor) has to be active during both stance and swing. Similarly, Levator-Depressor muscles are generally not in phase with swing and stance.. This variability is most probably due to the changes in kinematics, i.e., the spatial relation between legs and center of mass of the complete body. By introduction of two units representing explicitly the Stance and Swing the physical properties of the situation can be exploited without requiring specific explicit phase-coupling of the joint controllers for each kinematic situation. In addition, an architecture as depicted in Fig. 5 allows for easy simulation of the observation that different sensory input is exploited during swing and stance, including the Levator reflex and, not depicted in Fig. 5, the treading-on-tarsus (TOT) reflex (rule 6). Therefore, the question is open whether an explicit neuronal representation of the states Swing and Stance is necessary as is the case in Walknet or whether a decentralized solution is possible where Swing state and Stance state would occur as emergent properties? Such an alternative solution should, however, be able to control the movements of any leg type in any walking direction and to describe the experimental results as does Walknet.

*Control of backward walking and curve walking:* A related open area concerns the control of backward walking and curve walking. Forward walking can be elicited by tactile stimulation at the abdomen, for example. Backward walking can specifically be elicited by touching the antennae (Graham and Epstein 1985). There is only few data for backward walking in stick insects (Düsterhus and Schmitz 2009; Schmitz and Düsterhus 2009; Graham and Epstein 1985; Jeck and Cruse 2007; Akay et al. 2007; Rosenbaum et al. 2010). On the basis of application of Swing-net 2 or 3 a simple expansion has been discussed to allow simulation of backward walking by Schilling and Cruse (2008). Concerning the single-leg control, Tóth et al. (2012) proposed an extension to the model of Daun-Gruhn (2011) which, as Walknet, consists in representing the Swing and Stance states (see the paragraph above), there represented by the Protractor-Retractor CPG. However, in all models including Walknet, with respect to curve walking and backward walking a number of questions are still open, in particular questions concerning leg coordination and the determination of the AEP and PEP.

Concerning curve walking, information on how inter-leg coordination is changed is given by Dürr (2005). Furthermore, when negotiating curves, all legs performing side steps appear to contribute actively to the movement apart from the mechanical effects mentioned (Dürr 2005; Dürr and Ebeling 2005; Gruhn et al. 2009; Rosano and Webb 2007). Along these lines, Rosano and Webb (2007) introduced an expansion of Walknet, where front legs, middle legs and hind legs receive different influences: Front legs pull the body in the direction of the goal, middle legs pull/push the body sideways and hind legs rotate the body (in agreement with later experimental findings of Cruse et al. 2009). In addition, legs are assumed to be subject to a combination of velocity control and positive feedback. Simulating these assumptions showed good agreement with experimental data describing the body position of stick insects during turns. AEPs emerge in a similar way as has been discussed in Schumm and Cruse (2006). However, as for backward walking it remains open how PEPs are determined. Recently, Knops et al. (2013) proposed a model on curve walking. For a proof of concept, the authors simplified matters by using the front legs and hind legs as fixed, passive struts to support the body, while only middle legs are moved actively. The outer middle leg walks forward whereas the inner middle leg could move in different directions. Simulations were shown for two exemplar cases (side step or back step). However, as in the above-mentioned model, there is still no plausible idea as to how PEP or AEP might be determined during curve walking in a way as has been observed in the insects (Dürr 2005; Gruhn et al. 2009).

A specific subset of the models mentioned (Daun-Gruhn 2011; Daun-Gruhn et al. 2012; Tóth et al. 2012) shares the goal with our approach namely to be used as a tool helping to better understand the neuronal system responsible for the control of insect walking. The basic difference between both approaches concerns the detail to which the neuronal elements are represented. In the Walknet models, artificial neurons are applied that are characterized by piecewise linear activation functions (threshold, saturation) and that in some cases are equipped with dynamic properties (low-pass filter, which is the case for all motivation units, and the units used in the network developed by Schumm and Cruse 2006) and eventually using phasic (high-pass filter) properties. In contrast, models developed by Daun-Gruhn and coworkers operate with artificial neurons that are characterized by using Hodgkin–Huxley dynamics which implies the use of realistic synaptic connections. This approach allows to include specific neurons studied in either restrained or in some cases even free walking preparations [(e.g., Schmitz et al. 1991; Büschges et al. 1994; Kittmann et al. 1996; Uckermann and Büschges 2009)]. This approach is interesting as it allows a better representation of neurobiological reality, but at the same time is challenging as it requires to deal with many more free parameters including the fact that the neurons known to date may represent only a fraction of possibly relevant elements.

*Torque minimization, joint oscillators and flexibilitas cerea:* Only separately tested, but not tested in a complete version of Walknet are (i) the algorithm to approach a minimum torque sum (Lévy and Cruse 2008), and (ii) the neuronal representation of joint oscillators as proposed by Schumm and Cruse (2006). Flexibilitas cerea (Bässler 1983), an interesting property observed in non-active animals characterized by a leg joint showing an extremely slow return movement after a disturbance and interpreted as mimesis behavior, may be a property of the integral controller which is equipped with an extremely sensitive sensor monitoring angular velocity of the joints.

*Possible functional contribution of neuronal oscillators:* For each joint, there are groups of neurons being coupled to form networks (in Fig. 5 symbolized by blue units connected by mutual inhibition) that, in the deafferented situation and under the influence of pilocarpine or injected current show oscillating properties (e.g., Büschges et al. 1995), reviewed by Bässler and Büschges (1998). Under pilocarpine the oscillators show different eigenfrequencies. No coupling within legs, nor between legs, has been observed. These experiments do not provide direct information concerning the function of these central oscillators in the walking animal. Nonetheless, the most accepted interpretation is that these oscillators operate as generators controlling the rhythmic movements of the walking leg, which seems contrary to the assumption made for Walknet, where the (quasi-) rhythmic movement is controlled by sensory-driven controllers.

Is there a way to reconcile these seemingly opposing views? In a comprehensive review on “Central Pattern Generators” (CPG), Ijspeert (2008) listed five “interesting properties” and two “challenges” of central oscillators when they are used for the control of rhythmic motor output. In short, CPGs (i) allow for production of stable rhythms (return after a disturbance), (ii) are suited for distributed implementation, (iii) require few control parameters (concerning, e.g., speed, type of gaits), (iv) are suited to integrate sensory feedback and (v) form a substrate for learning. As two challenges, Ijspeert states that there is no sound design methodology yet and also no solid theoretical foundation for describing the properties of CPGs. But as these requirements are fulfilled also by sensory-driven controllers as is Walknet—and with respect to functionality they may in some aspects perform even better—it might be sensible to look how these two approaches are completing each other and how they might be integrated to improve the functionality of the complete system. It may well be possible that both principles are used for specific situations only. For example, in emergency situations, the central system may be used to replace the “sensory-driven oscillator.” A dramatic case of such an emergency could be the loss of one or several sensors due to an injury. A less dramatic case, but for biological systems probably equally important, occurs when fast rhythms are to be produced as is the case in a fast running insect. “Fast” is meant relative to the time delays resulting from the slow neuronal transduction. If sensory feedback is too slow, it may not be able to contribute to the production of the motor output required. Although such a central system might be inaccurate in the case of external disturbances, it may be better to use such a “quick but dirty” system as an approximation to the real world than wait for exact sensor readings that come too late. On the other hand, given a low enough walking speed, sensory-driven controllers are functionally better suited when walking in unpredictable environment.

Systems with mutual inhibitory connections, depending on the parameters chosen, may or may not produce cyclic activations. Such systems may also be used for functional purposes different from controlling ongoing rhythmic activity. As discussed by Cruse (2002) based on a simple model simulation, such networks may not be required to produce continuous oscillations to control the walking rhythm, but may only influence the next half-cycle (Schumm and Cruse 2006). For example, as discussed in this paper, parameters characterizing the actual stance movement (e.g., velocity and direction of foot trajectory) can influence the movement of the subsequent swing, but do not lead to continuous oscillations. In this way, the “predictive” property of this network is based on actual, local knowledge. Interestingly, it has been shown that such a network, when deafferented is able to generate motor patterns reminiscent of fictive motor activity, a central argument in favor of CPGs used to control the walking rhythm (Cruse 2002). It remains a matter of definition if such a network should be termed a central oscillator or not. In any case, a minor change of the parameters may result in a full fletched central oscillator.

To summarize, several types of models can be found in the literature able to control the three joints of a leg with possibly different phases and even different frequencies, a problem, as discussed above, less relevant for flying or swimming. (i) There are three coupled central oscillators with no or minor contribution of sensory input (e.g., Arena et al. 2004; Ijspeert 2008). (ii) There are three oscillators coupled via different sensory feedback (Beer and Gallagher 1992; Gallagher and Beer 1993; Tóth et al. 2012; Knops et al. 2013), and there are (iii) approaches where there are no central oscillators. These are the models of von Twickel et al. (2011) (being based on the model of Ekeberg et al. 2004), von Twickel et al. (2012), as well as most versions of Walknet. In Walknet, sensory coupling between leg joints shows some convergence (e.g., via PEP net and height controller) and divergence (e.g., from PEP net), whereas in the other models sensory coupling is represented by local reflexes mostly based on neurophysiological findings. All three types of models, pure oscillators, purely sensory-driven models and mixed models, appear to allow for production of sensible leg movements. Therefore, asking questions like which of solution (i) or (iii) is better suited to describe the biological results or shows better functional properties, may not be appropriate. Instead, a more interesting question for the future shall be how to combine both approaches to benefit from both of them, one aspect being that evolution has often implemented redundant control structures.

It might be mentioned in this context that, instead of using central oscillators as active devices to control motor output, they may also be used in a more passive way that is for predictive purposes, i.e., for sensory gating, or for filtering purposes via their bistability and hysteresis properties (von Twickel and Pasemann 2007). One way is to change sensory thresholds in a given time window (Degtyarenko et al. 1998). Moreover, central oscillators may be used on a longer time-scale to detect long-term deviations (e.g., in the case of sensory drift) by providing expectation values that could be compared with the sensory input. If a long-term deviation is detected, this information could be used to readjust the system via back-propagation or other learning algorithms (for example Kawato and Gomi 1992).

## Expansions beyond the level of insects

Walknet comprises a network architecture being characterized as a reactive system. This network based on a decentralized structure is able to autonomously control various behaviors that are able to adapt to a varying environment. The architecture can easily be expanded in different ways to cope with further behaviors, one example being the above-mentioned Navinet. This network allows for combining and selecting between different types of procedures as are path integration, context dependent landmark navigation, storage and retrieval of procedural memory and finding new shortcuts, as observed in ants and bees (Hoinville et al. 2012).

Most probably going beyond abilities of insects, Walknet has recently been equipped with a manipulable internal body model (Schilling et al. 2012; Schilling and Cruse 2007) and a small network allowing for top-down attention. The body model as such is used for sensor fusion purposes, i.e., to minimize errors arising in proprioceptive data, and for inverse kinematics. Using the body model as an inverse kinematic model, it can be used to control curve walking (Schilling et al. 2012) as well as backward walking. Both expansions, the body model and the attention system, together allow for identification and selection of, in the actual context, new behavioral elements that may serve the goal to solve an actual problem. A problem is defined by a situation that cannot be handled by Walknet. To this end, the internal body model is used to plan ahead, i.e., applied as a forward, predictive model. The capability to plan ahead has been proposed to define a system as to be a cognitive one (McFarland and Bösser 1993). Interestingly, this “cognitive expansion” of Walknet does not need a specific further network to represent any cognitive elements, but exploits the already given elements of the reactive network by “playing with different hypotheses” to find a solution. As this network allows for cognitive abilities by “parasiting” on the reactive structure, it has been called “reaCog” (Schilling and Cruse submitted; Cruse and Schilling 2010). In this way, Walknet, forming a reactive and embodied system, can serve as a starting point for further expansions introducing cognitive abilities as are planning ahead, imagined action, i.e., Freud’s (1911) “probehandeln” (Schilling and Cruse 2008), as well as introduction of metacognition and Theory of Mind (Cruse and Schilling 2011). Furthermore, there are proposals as to how reaCog might be equipped with linguistic properties (Cruse 2010).

## Footnotes

1 As long as we restrict ourselves to a description on the level of leg joints to be controlled and do not include possible movements in between body segments, not to mention descriptions on the muscular level or the neuronal level.

2 The output of Navinet (velocity, direction) can directly be used to control the corresponding parameters of Walknet. Therefore, both networks could be seen as modules of a larger system.

3 Such neuronal elements need not necessarily be realized by individual neurons as represented by the motivation units used here for simplicity, but may be represented by states of a larger neural network (e.g., Tani 2007).

4 Under such conditions gliding coordination is also observed in Walknet simulations (H. Cruse, not published).

## References

[CR1] Akay T, Ludwar BCh, Göritz ML, Schmitz J, Büschges A (2007). Segment specificity of load signal processing depends on walking direction in the stick insect leg muscle control system. J Neurosci.

[CR2] Annunziata S, Schneider A (2012) Physiologically based control laws featuring antagonistic muscle co-activation for stable compliant joint drives. Appl Bionics Biomech (published online). doi:10.3233/ABB-2012-0062

[CR3] Arena P, Fortuna L, Frasca M, Sicurella G (2004). An adaptive, self-organizing dynamical system for hierarchical control of bio-inspired locomotion. IEEE Trans Syst Man Cybern Part B.

[CR4] Ayers JL, Davis WJ (1977). Neuronal control of locomotion in the lobster, *Homarus americanus*. I. Motor programs for forward and backward walking. J Comp Physiol.

[CR5] Bartling C, Schmitz J (2000). Reaction to disturbances of a walking leg during stance. J Exp Biol.

[CR6] Bässler U (1972). Zur Beeinflussung der Bewegungsweise eines Beines von Carausius morosus durch Amputation anderer Beine. Kybernetik.

[CR7] Bässler U (1976). Reversal of a reflex to a single motoneuron in the stick insect *Carausius morosus*. Biol Cybern.

[CR8] Bässler U (1977). Sensory control of leg movement in the stick insect *Carausius morosus*. Biol Cybern.

[CR9] Bässler U (1979). Interactions of central and peripheral mechanisms during walking in first instar stick insects, *Extatosoma tiaratum*. Physiol Entomol.

[CR10] Bässler U (1983). Neural basis of elementary behavior in stick insects.

[CR11] Bässler U, Büschges A (1998). Pattern generation for stick insect walking movements-multisensory control of a locomotor program. Brain Res Rev.

[CR12] Bässler U, Rohrbacher J, Karg G, Breutel G (1991). Interruption of searching movements of partly restrained front legs of stick insects, a model situation for the start of a stance phase?. Biol Cybern.

[CR13] Bässler U, Dürner C, Fahrig T (1987). Motor output oscillations in denervated thoracic ganglia of walking stick insects. Zool Jb Physiol.

[CR14] Beer RD, Gallagher JC (1992). Evolving dynamical neural networks for adaptive behavior. Adapt Behav.

[CR15] Beer RD, Ritzmann RE, McKenna T (eds) (1993) Biological neural networks in invertebrate neuroethology and robotics. Academic Press, San Diego

[CR16] Beer RD, Quinn RD, Chiel HJ, Ritzmann RE (1997) Biologically inspired approaches to robotics. Commun ACM 40

[CR17] Bender JA, Simpson EM, Tietz BR, Daltorio KA, Quinn RD, Ritzmann RE (2011). Kinematic and behavioral evidence for a distinction between trotting and ambling gaits in the cockroach Blaberus discoidalis. J Exp Biol.

[CR18] Bläsing B (2006). Crossing large gaps: a simulation study of stick insect behaviour. Adapt Behav.

[CR19] Bläsing B, Cruse H (2004). Stick insect locomotion in a complex environment: climbing over large gaps. J Exp Biol.

[CR20] Bläsing B, Cruse H (2004). Mechanisms of stick insect locomotion in a gap crossing paradigm. J Comp Physiol A.

[CR21] Borgmann A, Scharstein H, Büschges A (2007). Intersegmental coordination: influence of a single walking leg on the neighboring segments in the stick insect walking system. J Neurophysiol.

[CR22] Borgmann A, Hooper S-L, Büschges A (2009). Sensory feedback induced by front-leg stepping entrains the activity of central pattern generators in caudal segments of the stick insect walking system. J Neurosci.

[CR23] Brunn EE, Dean J (1994). Intersegmental and local interneurons in the metathorax of the stick insect *Carausius morosus* that monitor middle leg position. J Neurophysiol.

[CR24] Buddenbrock  Wv (1921). Der Rhythmus der Schreitbewegungen der Stabheuschrecke Dixippus. Biol Zentralbl.

[CR25] Büschges A (1998). Inhibitory synaptic drive patterns motoneuronal activity in rhythmic preparations of isolated thoracic ganglia in the stick insect. Brain Res.

[CR26] Büschges A (1995). The role of local premotor interneurons in the generation of rhythmic motor activity in the stick insect. J Neurobiol.

[CR27] Büschges A, Kittmann R, Schmitz J (1994). Identified nonspiking interneurons in leg reflexes and during walking in the stick insect. J Comp Physiol A.

[CR28] Büschges A, Schmitz J, Bässler U (1995). Rhythmic patterns in the thoracic nerve cord of the stick insect induced by pilocarpine. J Exp Biol.

[CR29] Burkamp T (1996). Kontrolle der Phasenübergänge eines sechsbeinigen Laufsystems. Biologische Experimente und Computersimulation.

[CR30] Calvitti A, Beer RD (2000). Analysis of a distributed model of leg coordination i. Individual coordination mechanisms. Biol Cybern.

[CR31] Chasserat C, Clarac F (1980). Interlimb coordinating factors during driven walking in crustacea. J Comp Physiol.

[CR32] Clarac F, Cruse H (1982). Comparison of forces developed by the leg of the rock lobster when walking free or on a treadmill. Biol Cybern.

[CR33] Cruse H (1976). On the function of the legs in the free walking stick insect *Carausius morosus*. J Comp Physiol.

[CR34] Cruse H (1976). The control of the body position in the stick insect (*Carausius morosus*), when walking over uneven surfaces. Biol Cybern.

[CR35] Cruse H (1979). A new model describing the coordination pattern of the leg of a walking stick insect. Biol Cybern.

[CR36] Cruse H (1979). The control of the anterior extreme position of the hindleg of a walking insect. Physiol Entomol.

[CR37] Cruse H (1980). A quantitative model of walking incorporating central and peripheral influences. I. The control of the individual leg. Biol Cybern.

[CR38] Cruse H (1980). A quantitative model of walking incorporating central and peripheral influences. II. The connections between the different legs. Biol Cybern.

[CR39] Cruse H (1983). The influence of load and leg amputation upon coordination in walking crustaceans: a model calculation. Biol Cybern.

[CR40] Cruse H (1985). Coactivating influences between neighbouring legs in walking insects. J Exp Biol.

[CR41] Cruse H (1985). Which parameters control the leg movement of a walking insect? I. Velocity control during the stance phase. J Exp Biol.

[CR42] Cruse H (1985). Which parameters control the leg movement of a walking insect? II. The start of the swing phase. J Exp Biol.

[CR43] Cruse H (1990). What mechanisms coordinate leg movement in walking arthropods?. Trends Neurosci.

[CR44] Cruse H (2002). The functional sense of “central oscillations” in walking. Biol Cybern.

[CR45] Cruse H (2010) The talking stick: a cognitive system in a nutshell. In: Giuliani L (ed) Jahrbuch Wissenschaftskolleg zu, Berlin, pp 52–61

[CR46] Cruse H, Bartling C (1995). Movement of joint angles in the legs of a walking insect, *Carausius morosus*. J Insect Physiol.

[CR47] Cruse H, Bartling C, Cymbalyuk G, Dean J, Dreifert M (1995). A modular artificial neural net for controlling a six-legged walking system. Biol Cybern.

[CR48] Cruse H, Bartling C, Dean J, Kindermann T, Schmitz J, Schumm M, Wagner H (1996) Coordination in a six-legged walking system. Simple solutions to complex problems by exploitation of physical properties. In: Maes P, Mataric MJ, Meyer J-A, Pollack J, Wilson SW (eds) From animals to animats 4. The MIT Press/Bradford Books, Cambridge, pp 84–93

[CR49] Cruse H, Brunn D, Bartling Ch, Dean J, Dreifert M, Kindermann T, Schmitz J (1995). Walking—a complex behavior controlled by simple networks. Adapt Behav.

[CR50] Cruse H, Ehmanns I, Stübner S, Schmitz J (2009). Tight turns in stick insects. J Comp Physiol A.

[CR51] Cruse H, Epstein S (1982) Peripheral influences on the movement of the legs in a walking insect *Carausius morosus*. J Exp Biol 101:161–170

[CR52] Cruse H, Kindermann T, Schumm M, Dean J, Schmitz J (1998). Walknet—a biologically inspired network to control six-legged walking. Neural Netw.

[CR53] Cruse H, Knauth A (1989). Coupling mechanisms between the contralateral legs of a walking insect (*Carausius morosus*). J Exp Biol.

[CR54] Cruse H, Kühn S, Park S, Schmitz J (2004). Adaptive control for insect leg position: controller properties depend on substrate compliance. J Comp Physiol A.

[CR55] Cruse H, Müller U (1986). Two coupling mechanisms which determine the coordination of ipsilateral legs in the walking crayfish. J Exp Biol.

[CR56] Cruse H, Müller-Wilm U, Dean J (1993a) Artificial neural nets for controlling a 6-legged walking system. In: Meyer JA, Roitblat H, Wilson S (eds) From animals to animats, vol 2. MIT Press, Cambridge, pp 52–60

[CR57] Cruse H, Riemenschneider D, Stammer W (1989). Control of body position of a stick insect standing on uneven surfaces. Biol Cybern.

[CR58] Cruse H, Saxler G (1980). Oscillations of force in the standing legs of a walking insect (*Carausius morosus*). Biol Cybern.

[CR59] Cruse H, Schmitz J (1983). The control system of the femur tibia joint in the standing leg of a walking stick insect *Carausius morosus*. J Exp Biol.

[CR60] Cruse H, Schmitz J, Braun U, Schweins A (1993). Control of body height in a stick insect walking on a treadwheel. J Exp Biol.

[CR61] Cruse H, Schwarze W (1988). Mechanisms of coupling between the ipsilateral legs of a walking insect (*Carausius morosus*). J Exp Biol.

[CR62] Cruse H, Silva Saavedra GM (1996). Curve walking in crayfish. J Exp Biol.

[CR63] Cruse H, Schilling M (2010) Getting cognitive. In: Bläsing B, Puttke M, Schack T (eds) The neurocognition of dance. Psychology Press, London, pp 53–74

[CR64] Cruse H, Schilling M (2011) From egocentric systems to systems allowing for theory of mind and mutualism. In: Lenaerts T, Giacobini M, Bersini H, Bourgine P, Dorigo M, Doursat R (eds) Advances in artificial life, ECAL 2011, proceedings of the eleventh European conference on the synthesis and simulation of living systems. MIT Press, Cambridge, pp 185–192

[CR65] Cruse H, Wehner R (2011). No need for a cognitive map: decentralized memory for insect navigation. PLoS Comput Biol.

[CR66] Cruse H, Schilling M (2013) How and to what end may consciousness contribute to action? Attributing properties of consciousness to an embodied, minimally cognitive artificial neural network. Front Psychol 4:324. doi:10.3389/fpsyg.2013.00324 10.3389/fpsyg.2013.00324 PMC3684785 23785343

[CR67] Daun-Gruhn S (2011). A mathematical modeling study of inter-segmental coordination during stick insect walking. J Comp Neurosci.

[CR68] Daun-Gruhn S, Büschges A (2011). From neuron to behavior: dynamic equation-based prediction of biological processes in motor control. Biol Cybern.

[CR69] Daun-Gruhn S, Tóth TI, Borgmann A (2012). Dominance of local sensory signals over inter-segmental effects in a motor system: modeling studies. Biol Cybern.

[CR70] Dean J (1984). Control of leg protraction in the stick insect: a targeted movement showing compensation for externally applied forces. J Comp Physiol A.

[CR71] Dean J (1990). Coding proprioceptive information to control movement to a target: simulation with a simple neural network. Biol Cybern.

[CR72] Dean J (1991). A model of leg coordination in the stick insect, *Carausius morosus*. I. A geometrical consideration of contralateral and ipsilateral coordination mechanisms between two adjacent legs. Biol Cybern.

[CR73] Dean J (1991). A model of leg coordination in the stick insect, *Carausius morosus*. II. Description of the kinematic model and simulation of normal step patterns. Biol Cybern.

[CR74] Dean J (1992). A model of leg coordination in the stick insect, *Carausius morosus*. III. Responses to perturbations of normal coordination. Biol Cybern.

[CR75] Dean J (1992). A model of leg coordination in the stick insect, *Carausius morosus*. IV. Comparisons of different forms of coordinating mechanismus. Biol Cybern.

[CR76] Dean J, Wendler G (1982). Stick insects walking on a wheel: perturbations induced by obstruction of leg protraction. J Comp Physiol.

[CR77] Dean J, Wendler G (1983). Stick insect locomotion on a walking wheel: interleg coordination of leg position. J Exp Biol.

[CR78] Degtyarenko AM, Simon ES, Norden-Krichmar T, Burke RE (1998). Modulation of oligosynaptic cutaneous and muscle afferent reflex pathways during fictive locomotion and scratching in the cat. J Neurophysiol.

[CR79] Diederich B, Schumm M, Cruse H (2002). Stick insects walking along inclined surfaces. Integr Comp Biol.

[CR80] Dürr V (2001). Stereotypic leg searching-movements in the stick insect: kinematic analysis, behavioural context and simulation. J Exp Biol.

[CR81] Dürr V (2005). Context-dependent changes in strength and efficacy of leg coordination mechanisms. J Exp Biol.

[CR82] Dürr V, Ebeling W (2005). The behavioural transition from straight to curve walking: kinetics of leg movement parameters and the initiation of turning. J Exp Biol.

[CR83] Dürr V, Schmitz J, Cruse H (2004). Behaviour-based modelling of hexapod locomotion: linking biology and technical application. Arthropod Struct Dev.

[CR84] Düsterhus D, Schmitz J (2009) Sensory control of backward walking in the stick insect: I. Intrasegmental influences. Proc. Soc. Neuroscience Meeting, Chicago, USA

[CR85] Ebeling W, Dürr V (2006). Perturbation of leg protraction causes context-dependent modulation of inter-leg coordination, but not of avoidance reflexes. J Exp Biol.

[CR86] Ekeberg O, Blümel M, Büschges A (2004). Dynamic simulation of insect walking. Arthropod Struct Dev.

[CR87] Espenschied KS, Quinn RD, Chiel HJ, Beer RD (1993). Leg coordination mechanisms in the stick insect applied to hexapod robot locomotion. Adapt Behav.

[CR88] Ferrell C (1995). A comparison of three insect.-inspired locomotion controllers. Robot Auton Syst.

[CR89] Flannigan WC, Nelson GM, Quinn RD (1998) Locomotion controller for a crab-like robot. In: IEEE proceedings of the robotics and automation 1998, Leuven, Belgium, pp 152–162

[CR90] Foth E, Bässler U (1985). Leg movements of stick insects walking with five legs on a treadwheel and with one leg on a motor-driven belt. 1. General results and 1:1-coordination. Biol Cybern.

[CR91] Freud S (1911) Formulierung über die zwei Prinzipien des psychischen Geschehens. In Gesammelte Werke, Bd. VIII, pp 229–238

[CR92] Frik M, Guddat M Karatas, M Losch CD (1999) A novel approach to autonomous control of walking machines In: Proceedings of the 2nd conference on climbing and walking robots, CLAWAR 1999. Professional Engineering Publishing, Bury St. Edmunds, UK, pp 333–342

[CR93] Gallagher JC, Beer RD, Meyer JA, Roitblat H, Wilson S (1993). A qualitative dynamical analysis of evolved locomotion controllers. From animals to animats 2.

[CR94] Grabowska M, Godlewska E, Schmidt J, Daun-Gruhn S (2012). Quadrupedal gaits in hexapod animals—stepping patterns in free-walking adult stick insects. J Exp Biol.

[CR95] Graham D (1972). A behavioural analysis of the temporal organisation of walking movements in the 1st instar and adult stick insect. J Comp Physiol.

[CR96] Graham D (1977). The effect of amputation and leg restraint on the free walking coordination of the stick insect *Carausius morosus*. J Comp Physiol.

[CR97] Graham D (1979). Effects of circum oesophageal lesion on the behaviour of the stick insect Carausius. II. Changes in walking coordination. Biol Cybern.

[CR98] Graham D (1985). Pattern and control of walking in insects. Adv Insect Physiol.

[CR99] Graham D, Cruse H (1981). Coordinated walking of stick insects on a mercury surface. J Exp Biol.

[CR100] Graham D, Epstein D (1985). Behaviour and motor output for an insect walking on a slippery surface. II. Backward walking. J Exp Biol.

[CR101] Graham D, Wendler G (1981). Motor output to the protractor and retractor muscle in a stick insect walking on a treadwheel. Physiol Entomol.

[CR102] Gruhn M, Zehl L, Büschges A (2009). Straight walking and turning on a slippery surface. J Exp Biol.

[CR103] Hassenstein B (1983). Funktionsschaltbilder als Hilfsmittel zur Darstellung theoretischer Konzepte in der Verhaltensbiologie. Zool Jb Physiol.

[CR104] Hoinville T, Wehner R, Cruse H (2012) Learning and retrieval of memory elements in a navigation task. Living Machines Conf. Barcelona, pp 120–131

[CR105] Hooper SL, Guschlbauer C, Blümel M, Rosenbaum P, Gruhn M, Akay T, Büschges A (2009). Neural control of unloaded leg posture and of leg swing in stick insect, cockroach, and mouse differs from that in larger animals. J Neurosci.

[CR106] Hughes GM (1952). The co-ordination of insect movements. I. The walking movements of insects. J Exp Biol.

[CR107] Ijspeert A (2008). Central pattern generators for locomotion control in animals and robots: a review. Neural Netw.

[CR108] Jeck T, Cruse H (2007). Walking in Aretaon asperrimus. J Insect Physiol.

[CR109] Karg G, Breutel G, Bässler U (1991). Sensory influences on the coordination of two leg joints during searching movements of stick insects. Biol Cybern.

[CR110] Kawato M, Gomi H (1992). The cerebellum and VOR/OKR learning-models. Trends Neurosci.

[CR111] Kindermann T (2002). Behavior and adaptability of a six-legged walking system with highly distributed control. Adapt Behav.

[CR112] Kittmann R, Schmitz J, Büschges A (1996). Premotor interneurons in generation of adaptive leg reflexes and voluntary movements in stick insects. J Neurobiol.

[CR113] Knops S, Tóth TI, Guschlbauer C, Gruhn M, Daun-Gruhn S (2013). A neuro- mechanical model for curve walking in the stick insect. J Neuroph.

[CR114] Lévy J (2009). Controlling a system with redundant degrees of freedom: transition from standing to walking. J Comp Physiol A.

[CR115] Lévy J, Cruse H (2008). Controlling a system with redundant degrees of freedom: I. Torque distribution in still standing stick insects. J Comp Physiol A.

[CR116] Lévy J, Cruse H (2008). Controlling a system with redundant degrees of freedom: II. Solution of the force distribution problem without a body model. J Comp Physiol A.

[CR117] Maes P, Meyer JA, Wilson S (1991). A bottom-up mechanism for behavior selection in an artificial creature. From animals to animats.

[CR118] Matheson T, Dürr V (2003). Load compensation in targeted limb movements of an insect. J Exp Biol.

[CR119] McFarland D, Bösser T (1993) Intelligent behavior in animals and robots. MIT Press, Cambridge

[CR120] Müller U, Cruse H (1991). The contralateral coordination of walking in the crayfish *Astacus leptodactylus*. II. Model calculations. Biol Cybern.

[CR121] Müller-Wilm U, Dean J, Cruse H, Weidemann HJ, Eltze J, Pfeiffer F (1992). Kinematic model of stick insect as an example of a 6-legged walking system. Adapt Behav.

[CR122] Neuser K, Triphan T, Mronz M, Poeck B, Strauss R (2008). Analysis of a spatial orientation memory on Drosophila. Nature.

[CR123] Paskarbeit J, Schmitz J, Schilling M, Schneider A (2010) Layout and construction of a hexapod robot with increased mobility. In: Proceedings of the 3rd IEEE RAS/EMBS international conference on biomedical robotics and biomechatronics (IEEE BIOROB 2010), September 26–29, 2010. Tokyo, Japan, pp 621–625

[CR124] Pearson KG (1972). Central programming and reflex control of walking in the cockroach. J Exp Biol.

[CR125] Pearson KG, Franklin R (1984). Characteristics of leg movements and patterns of coordination in locusts walking on rough terrain. Int J Robot Res.

[CR126] Pearson KG, Iles JF (1973). Nervous mechanisms underlying intersegmental coordination of leg movements during walking in cockroach. J Exp Biol.

[CR127] Pfeiffer F, Eltze J, Weidemann HJ (1995). Six-legged technical walking considering biological principles. Robot Auton Syst.

[CR128] Ritzmann RE, Büschges A (2007) Adaptive motor behavior in insects. Curr Opin Neurobiol 17(6):629–36. Epub 2008 Mar 4. Review 10.1016/j.conb.2008.01.001 18308559

[CR129] Rosano H, Webb B (2007). A dynamic model of thoracic differentiation for the control of turning in the stick insect. Biol Cybern.

[CR130] Rosenbaum P, Wosnitza A, Büschges A, Gruhn M (2010). Activity patterns and timing of muscle activity in the forward walking and backward walking stick insect *Carausius morosus*. J Neurophysiol.

[CR131] Schilling M (2011) Universally manipulable body models—dual quaternion representations in layered and dynamic MMCs. Auton Robot 30(4):399–425

[CR132] Schilling M, Cruse H (2007) Hierarchical MMC networks as a manipulable body model. In: Proceedings of the international joint conference on neural networks (IJCNN), Orlando, FL, pp 2141–2146

[CR133] Schilling M, Cruse H (2008) The evolution of cognition—from first order to second order embodiment. In: Wachsmuth I, Knoblich G (eds) Modeling communication with robots and virtual humans. Lecture Notes in Artificial Intelligence. Springer, Berlin, pp 77-108

[CR134] Schilling M, Cruse H (submitted) reaCog, a minimal cognitive controller based on recruitment of reactive systems 10.3389/fnbot.2017.00003 PMC5276858 28194106

[CR135] Schilling M, Cruse H, Arena P (2007) Hexapod Walking: an expansion to Walknet dealing with leg amputations and force oscillations. Biol Cybern 96:323–340 10.1007/s00422-006-0117-1 17106698

[CR136] Schilling M, Paskarbeit J, Schmitz J, Schneider A, Cruse H (2012) Grounding an internal body model of a hexapod walker—control of curve walking in a biological inspired robot. In: Proceedings of IEEE/RSJ international conference on intelligent robots and systems, IROS 2012, pp 2762–2768

[CR137] Schilling M, Paskarbeit J, Hüffmeier A, Schneider A, Schmitz J, Cruse H (submitted) A hexapod walker using a heterarchical architecture for action selection 10.3389/fncom.2013.00126 PMC3774992 24062682

[CR138] Schmitz J, Gewecke M, Wendler G (1985). Control of the leg joints in stick insects: differences in the reflex properties between the standing and the walking states. Insect locomotion.

[CR139] Schmitz J (1986). The depressor trochanteris motoneurones and their role in the coxo-trochanteral feedback loop in the stick insect *Carausius morosus*. Biol Cybern.

[CR140] Schmitz J (1986). Properties of the feedback system controlling the coxa-trochanter joint in the stick insect *Carausius morosus*. Biol Cybern.

[CR141] Schmitz J (1993). Load compensatory reactions in the proximal leg joints of stick insects during standing and walking. J Exp Biol.

[CR142] Schmitz J, Bartling C, Brunn DE, Cruse H, Dean J, Kindermann T, Schumm M, Wagner H (1995). Adaptive properties of ”hard-wired” neuronal systems. Verh Dtsch Zool Ges.

[CR143] Schmitz J, Büschges A, Kittmann R (1991). Intracellular recordings from nonspiking interneurons in a semiintact, tethered walking insect. J Neurobiol.

[CR144] Schmitz J, Düsterhus D (2009) Sensory control of backward walking in the stick insect: II. Intersegmental influences. Proc Soc Neuroscience Meeting Chicago, USA

[CR145] Schmitz J, Hassfeld G (1989). The treading-on-tarsus reflex in stick insects: phase-dependence and modifications of the motor output during walking. J Exp Biol.

[CR146] Schmitz J, Schneider A, Schilling M, Cruse H (2008). No need for a body model: positive velocity feedback for the control of an 18-DOF robot walker. Appl Bionics Biomechs.

[CR147] Schmitz J, Stein W (2000). Convergence of load and movement information onto leg motoneurons in insects. J Neurobiol.

[CR148] Schmitz J, von Kamp A, Kindermann T, Cruse H (1999) Adaptations to increased load in a control system governing movements of biological and artificial walking machines. In: Blickhan R, Wisser A, Nachtigall W (eds) IONA report 13: motion systems. Elsevier, Amsterdam, pp 50–51

[CR149] Schneider A, Cruse H, Fischer B, Schmitz J (2007) A self-adjusting negative feedback joint controller for legs standing on moving substrates of unknown compliance. In: Proceedings of SPIE 6592, bioengineered and bioinspired systems III, 65920M (May 22, 2007). doi:10.1117/12.721723

[CR150] Schneider A, Cruse H, Schmitz J (2008). Winching up heavy loads with a compliant arm: a new local joint controller. Biol Cybern.

[CR151] Schneider A, Paskarbeit J, Schäffersmann M, Schmitz J (2011) Biomechatronics for embodied intelligence of an insectoid robot. Proc ICRA (2)’11, pp 1–11

[CR152] Schumm M, Cruse H (2006). Control of swing movement: influences of differently shaped substrate. J Comp Physiol A.

[CR153] Steingrube S, Timme M, Wörgötter F, Manoonpong P (2010). Self-organized adaptation of a simple neural circuit enables complex robot behaviour. Nat Phys.

[CR154] Strauss R (2002). The central complex and the genetic dissection of locomotor behavior. Curr Opin Neurobiol.

[CR155] Tani J (2007). On the interactions between top-down anticipation and bottom-up regression. Front Neurorobot.

[CR156] Theunissen LM, Bekemeier HH, Dürr V (2012) On the natural statistics of insect locomotion: indications for two distinct mechanisms of step generation. Front Behav Neurosci Conference Abstract: Tenth International Congress of Neuroethology. doi:10.3389/conf.fnbeh.2012.27.00150

[CR157] Tóth TI, Knops S, Daun-Gruhn S (2012). A neuro-mechanical model explaining forward and backward stepping in the stick insect. J Neurophys.

[CR158] von Holst E (1939). Die relative Koordination als Phänomen und als Methode zentralnervöser Funktionsanalyse. Erg Physiol.

[CR159] von Holst E (1943). Über relative Koordination bei Arthropoden. Pflügers Arch.

[CR160] von Twickel A, Hild M, Siedel T, Patel V, Pasemann F (2012). Neural control of a modular multi-legged walking machine: simulation and hardware. Robot Auton Syst.

[CR161] von Twickel A, Büschges A, Pasemann F (2011). Deriving neural network controllers from neuro-biological data: implementation of a single-leg stick insect controller. Biol Cybern.

[CR162] von Twickel A, Pasemann F (2007). Reflex-oscillations in evolved single leg neurocontrollers for walking machines. Nat Comput.

[CR163] von Uckermann G, Büschges A (2009) Premotor interneurons in the local control of stepping motor output for the stick insect single middle leg. J Neurophysiol 102:1956–1975 10.1152/jn.00312.2009 19605613

[CR164] Wendler G (1964). Laufen und Stehen der Stabheuschrecke: Sinnesborsten in den Beingelenken als Glieder von Regelkreisen. Z vergl Physiol.

[CR165] Wendler G (1968) Ein Analogmodell der Beinbewegungen eines laufenden Insekts. In: Kybernetik 1968. Beihefte zu “Elektronische Rechenanlagen” vol 18, R.Oldenbourg Verlag, München, Wien, pp 68–74

[CR166] Wildmann M, Ott S, Burrows M (2002) GABA-like immunoreactivity in nospiking interneurons of the locust metathoracic ganglion. J Exp Biol 205:3651–3659 10.1242/jeb.205.23.3651 12409491

[CR167] Wilson DM (1966). Insect walking. Annu Rev Entomol.

[CR168] Wolpert DM, Ghahramani Z, Flanagan JR (2001). Perspectives and problems in motor learning. Trends Cogn Sci.

[CR169] Wosnitza A, Bockemühl T, Dübbert M, Scholz H, Büschges A (2013). Interleg coordination in the control of walking speed in Drosophila. J Exp Biol.

